# Navigating cell maps by deep learning integration of single-cell and spatially resolved transcriptomics

**DOI:** 10.1093/bib/bbag404

**Published:** 2026-07-30

**Authors:** Yanan Chen, Ruoyu Chen, Shaoqiang Zhang, Yong Chen

**Affiliations:** Department of Data Science, College of Computer and Information Engineering, Tianjin Normal University, 393 Binshui West Road, Xiqing District, Tianjin, Tianjin 300387, China; Department of Physiology, College of Arts and Sciences, University of Pennsylvania, 3600 Market Street, Philadelphia, PA 19104, United States; Department of Data Science, College of Computer and Information Engineering, Tianjin Normal University, 393 Binshui West Road, Xiqing District, Tianjin, Tianjin 300387, China; Department of Biological and Biomedical Sciences, College of Science and Mathematics, Rowan University, 201 Mullica Hill Road, Glassboro, NJ 08028, United States

**Keywords:** spatially resolved transcriptomics, scRNA-seq, unsupervised deep learning, graph attention autoencoder, data integration

## Abstract

Single-cell RNA sequencing (scRNA-seq) enables genome-wide gene expression profiling at single-cell resolution but loses the spatial context essential for interpreting cell identity and tissue organization. In contrast, spatially resolved transcriptomics (SRT) preserves spatial information but typically lacks single-cell resolution or complete transcriptome coverage. To obtain a more comprehensive view of heterogeneous spatial domains and cellular gene expression, we present Cell2Map, an unsupervised deep learning method that integrates scRNA-seq and SRT data from the same tissue region. Cell2Map assigns individual cells to SRT spots using a graph attention autoencoder equipped with a specially designed multi-term objective function that jointly optimizes expression-based, density-based, and embedding-level similarity and distance constraints. On benchmark datasets from mouse cerebellum and hippocampus, Cell2Map achieves higher single-cell mapping precision and overall accuracy than three popular methods (Celloc, CytoSPACE, Tangram) across a range of noise levels and spot cell densities. In real cancer applications, Cell2Map resolves intratumoral heterogeneity by accurately localizing tumor subclones and separating normal epithelial cells from ductal carcinoma *in situ* regions, and more faithfully reconstructs tumor microenvironments and immune-cell localization than competing approaches. Across breast cancer and myocardial infarction datasets, Cell2Map consistently attains higher sensitivity with fewer false positives, in close agreement with histological and biological annotations.

## Introduction

Single-cell sequencing technologies have transformed our ability to dissect the complexity of biological systems over the past decade. Among them, single-cell RNA sequencing (scRNA-seq) provides genome-wide gene expression profiles at single-cell resolution, enabling characterization of cell types, functional states, developmental trajectories, and disease progression within tissues. However, scRNA-seq requires enzymatic digestion or mechanical dissociation of tissues, which disrupts the spatial context that is critical for understanding cell states, cellular niches, and microenvironmental interactions [1–3]. Spatially resolved transcriptomics (SRT) technologies complement scRNA-seq by preserving positional information in tissue sections. Sequencing-based platforms such as 10x Visium [4], Slide-seq [5], and Stereo-seq [6] provide transcriptome-wide coverage but typically at multicellular or subcellular spot resolution, whereas image-based approaches, including MERFISH [7], smFISH [8], seqFISH+ [9], and STARmap [10], achieve near single-cell or subcellular resolution but are limited in the number of profiled genes. Thus, there is an inherent trade-off between spatial resolution and transcriptome breadth. Computational integration of SRT with scRNA-seq data is therefore essential to bridge these complementary strengths, improve cell-type and biomarker identification, and gain a more comprehensive view of tissue architecture in health and disease [11–13]. Even with recently developed high-resolution platforms such as Visium HD (~2 μm resolution) [14], integration with scRNA-seq remains valuable because different protocols capture distinct aspects of transcriptional and technical variation [15].

A large number of existing methods, such as Cell2location [16], SpatialDWLS [17], SpaDecon [18], SpatialScope [19], CARD [20], DSTG [21], SpatialcoGCN [22], and LETSmix [23], focus primarily on deconvolving SRT spots into cell-type proportions rather than explicitly assigning individual scRNA-seq data to spatial locations. While these approaches improve cell-type composition estimates, they do not provide single-cell resolution maps and therefore offer limited support for downstream analyses, such as single-cell clustering, lineage tracing or cell-cell communication [24]. To address this, several recent methods, including Tangram [25], CellTrek [26], CytoSPACE [27], and Celloc [28], attempt to map individual scRNA-seq cells to SRT spots. For example, Tangram enforces consistency between predicted cell density and gene expression profiles at each spatial spot, whereas CytoSPACE further constrains cell-type proportions during mapping. In contrast, Celloc incorporates an autoencoder-based strategy to learn latent representations and applies a correlation-based scoring strategy within the embedding space to guide cell-to-spot assignment. Related models such as CeLEry [29] and CellContrast [30] instead infer spatial coordinates for scRNA-seq cells via supervised learning or spatial smoothness assumptions, but they do not perform explicit one-to-one cell-to-spot mapping. Technically, they are not designed for data integration analysis, but for spatial information inference of cells in scRNA-seq data by training the relationship between gene expression profiles and spatial locations based on a large number of supervised profile-to-location samples or based on the assumption that spatially proximate cells exhibit similar expression. Although Tangram, CytoSPACE, and Celloc can perform single cell-to-spot mapping by optimizing different objective functions to estimate mapping matrices from single cells to spatial spots, their objective functions typically capture only limited or partial features during optimization. For example, Tangram’s objective emphasizes consistency in cell density and gene expression within each spot before and after mapping, whereas CytoSPACE additionally incorporates the fractional abundance of each cell type in the spatial transcriptomics data. Celloc, in turn, uses a mapping score based on Pearson correlation in an embedding space. Therefore, a promising direction is to jointly optimize multiple complementary criteria, such as distance- and correlation-based measures in both the original expression space and learned latent spaces, to improve mapping accuracy and robustness [13, 15].

Here, we introduce Cell2Map, a new deep learning framework that integrates scRNA-seq and SRT data to achieve flexible, high-resolution cell-to-spot assignment. Cell2Map builds on a graph attention autoencoder (GATE) to learn low-dimensional embeddings of both scRNA-seq cells and SRT spots, using graphs constructed from spatial adjacency (for SRT) and expression similarity (for scRNA-seq). On top of these embeddings, Cell2Map optimizes a mapping matrix via a multi-term objective function that combines density, correlation, and distance-based terms in both expression and latent spaces. This design enables Cell2Map to leverage complementary information from scRNA-seq and SRT data while improving mapping accuracy and robustness across diverse experimental settings. On simulated mouse cerebellum and hippocampus benchmarks, Cell2Map outperforms Celloc, CytoSPACE, and Tangram in mapping precision and overall accuracy across varying noise levels and spot cell densities. In real datasets [breast cancer and myocardial infarction (MI)], Cell2Map better resolves tissue heterogeneity and cell-type localization with higher sensitivity and fewer false positives, consistent with histological and biological annotations.

## Materials and methods

### Overview of Cell2Map

**Figure 1:**
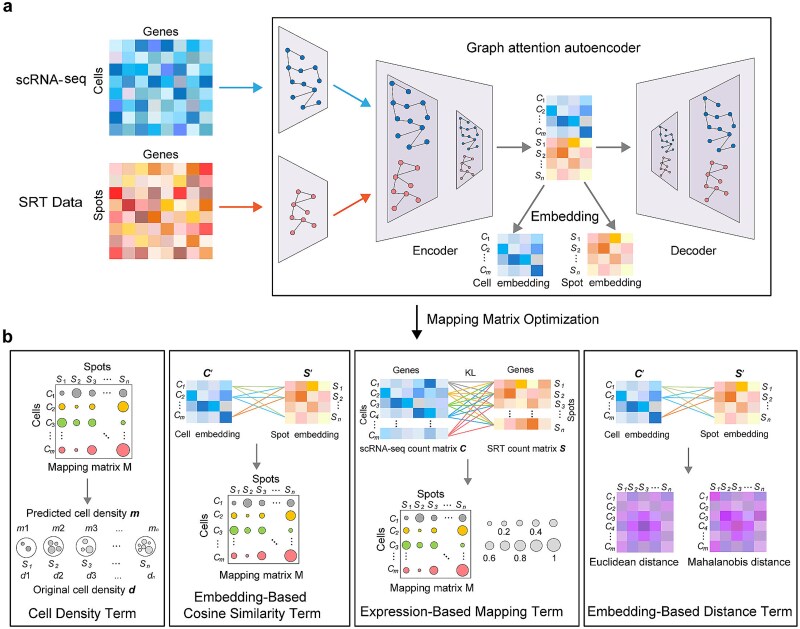
Cell2Map framework for mapping single cells in scRNA-seq to spatial transcriptomics spots; (a) Cell2Map integrates scRNA-seq and SRT data using a GATE to learn low-dimensional embeddings; modality-aligned embeddings for spatial spots and individual cells; (b) the cell-to-spot mapping matrix is estimated jointly by optimizing an objective function comprising four complementary constraints.

To leverage complementary spatial and transcriptional information to improve mapping accuracy and stability, Cell2Map maps scRNA-seq cells to SRT spots by jointly modeling the two modalities with a GATE (Fig. 1a). Specifically, Cell2Map constructs two graphs from matched tissue samples: an SRT spot graph, where nodes are spots connected by spatial adjacency and an scRNA-seq cell graph, where nodes are cells connected based on gene-expression similarity. These graphs are encoded and decoded within a shared GATE framework to learn low-dimensional embeddings for both cells and spots, while simultaneously optimizing an unknown cell-to-spot mapping matrix $M$.

To estimate $M$robustly, Cell2Map employs a four-term objective function that combines four complementary constraints (Fig. 1b). It retains an expression-based mapping term (MT) to align mapped spot-level expression with observed SRT profiles and introduces three additional terms to capture information that is not fully represented by expression alignment alone. Two of these constraints are computed in the latent embedding space to reduce computational complexity and improve robustness: (i) a cosine-similarity term that encourages high correlation between mapped cells and spots, and (ii) an embedding-distance term (DiT) (e.g. Euclidean and Mahalanobis) that promotes proximity between matched cell and spot embeddings. Together with a cell-density term that regularizes cell allocation across spots, these four terms jointly guide $M$ toward accurate and stable cell-to-spot assignments across diverse settings.

### Data preprocessing and preparation

The Python package SCANPY (version1.9.6) [31] was used for preprocessing and filtering the raw scRNA-seq data and SRT data from the same tissue sample. Specifically, we (a) removed genes expressed in fewer than one cell or spot, (b) filtered out low-quality cells and spots expressing fewer than three genes, (c) normalized counts to counts per million using “scanpy.pp.normalize_total(data, target_num = 1e6)” and applied log-transformation with “scanpy.pp.log1p(),” and (d) retained the intersection of genes shared by both modalities. After preprocessing, we obtained two aligned expression matrices $\boldsymbol{C}\in{\mathbb{R}}^{m\times g}$ and $\boldsymbol{S}\in{\mathbb{R}}^{n\times g}$, representing gene expression profiles for $m$ cells, $n$ spatial spots and $g$ genes.

To incorporate spot-level cell density information, we estimated the number of cells per spot $\boldsymbol{s}={\left({s}_1,{s}_2,\dots, {s}_n\right)}^{\mathrm{T}}$ from Hematoxylin and Eosin (H&E) images and derived the normalized density vector $\boldsymbol{d}={\left({d}_1,{d}_2,\dots, {d}_n\right)}^{\mathrm{T}}$, where ${d}_j=\frac{s_j}{\sum_{i=1}^n{s}_j}$. StarDist [32] is used for nucleus segmentation on H&E images to count cells per spot. When H&E-based estimates are unavailable, we default to setting a uniform cell density of each spot to $1/n$.

The goal of Cell2Map is to obtain an optimal mapping matrix $\boldsymbol{M}={\left({M}_{ij}\right)}_{m\times n}\in{\mathbb{R}}^{m\times n}$ from $m$ cells to $n$ spots, where ${M}_{ij}$ is the probability of the $i$-th cell being within the $j$-th spot, representing a soft assignment of the $i$-th cell to the $j$-th spot. The mapping satisfies the normalization constraint $\sum_{j=1}^n{M}_{ij}=1$ for each cell $i.$ For downstream analyses requiring discrete assignments, the soft mapping can be converted to hard assignments by assigning each cell to the spot with the highest probability.

### Graph attention autoencoder

Cell2Map employs a GATE to learn low-dimensional latent representations from the two preprocessed matrices $\boldsymbol{C}$ and $\boldsymbol{S}$. To ensure that the low-dimensional latent representations of single cell and spot expression profiles are embedded in the same embedding space, we concatenate $\boldsymbol{C}$ and $\boldsymbol{S}$, and then input them into the same GATE, which consists of an encoder and a decoder.

For scRNA-seq data, the KNN (k-nearest neighbors) algorithm is employed to construct a KNN graph, in which each node has $k$ nearest nodes based on expression profiles. For SRT data, the cKDTree method (the “scipy.spatial.cKDTree” class) [33] is used to construct a neighbor graph for all nodes (spots) in a given spatial radius. The two graphs are merged and saved as an adjacency matrix.

In each layer of the encoder, the hidden representation of each node is aggregated from the features of its neighboring nodes. To learn hidden representations of each node, we need to combine the node features with the graph structure in each hidden embedding space. To measure the importance of various neighbors, different weights are adaptively assigned to edges incident with different neighbors. Formula (1) is used to compute the representation of node $i$ in the $l$-th encoder layer.


(1) \begin{eqnarray*} {\boldsymbol{z}}_i^{(l)}=\sum_{j\in{\boldsymbol{N}}_i}{\alpha}_{ij}^{(l)}\sigma \left({\boldsymbol{W}}^{(l)}{\boldsymbol{z}}_j^{\left(l-1\right)}\right) \end{eqnarray*}

Here, ${\boldsymbol{z}}_i^{(l)}$denotes the output representation of node $i$, ${\boldsymbol{N}}_i$ denotes the neighbors of node $i$, ${\boldsymbol{W}}^{(l)}$is the trainable weight matrix, ${\alpha}_{ij}^{(l)}$ denotes the attention coefficient indicating the importance of neighbor node $j$ to node $i$, and $\sigma$is a nonlinearity activation function (here Exponential Linear Unit is used). Note that the initial node representations ${\boldsymbol{z}}_i^{(0)},i=1,2,\dots, m+n$ are the preprocessed expression profiles of cells or spots.

To calculate the attention coefficient ${\alpha}_{ij}^{(l)}$, we first measure the importance of neighbor node $j$ to node $i$, represented by ${\gamma}_{ij}^{(l)}$, and then normalize across all neighbor nodes $j\in{\boldsymbol{N}}_i$ with a softmax function to make them comparable easily across nodes. The calculation functions for ${\gamma}_{ij}^{(l)}$ and ${\alpha}_{ij}^{(l)}$ are shown in Formulas (2) and (3), respectively.


(2) \begin{eqnarray*} {\gamma}_{ij}^{(l)}=\mathrm{Sigmoid}\left({\boldsymbol{V}}^{(l)^T}\delta \left({\boldsymbol{W}}^{(l)}{\boldsymbol{z}}_i^{\left(l-1\right)}\right)+{\boldsymbol{\varLambda}}^{(l)^T}\delta \left({\boldsymbol{W}}^{(l)}{z}_j^{\left(l-1\right)}\right)\ \right) \end{eqnarray*}

(3) \begin{eqnarray*} {\alpha}_{ij}^{(l)}=\mathrm{Softmax}\left({\gamma}_{ij}^{(l)}\right)=\frac{\exp \left({\gamma}_{ij}^{(l)}\right)}{\sum_{k\in{N}_i}\exp \left({\gamma}_{ik}^{(l)}\right)} \end{eqnarray*}

In Formula (2), ${\boldsymbol{W}}^{(l)}\in{\mathbb{R}}^{d^{(l)}\times{d}^{\left(l-1\right)}}$, ${\boldsymbol{V}}^{(l)}\in{\mathbb{R}}^{d^{(l)}}$, and ${\boldsymbol{\varLambda}}^{(l)}\in{\mathbb{R}}^{d^{(l)}}$are trainable weights, where ${d}^{(l)}$ is the dimension of node representations in the $l$-th layer, and $\delta$ is an activation function.

The encoder consists of $L$ layers, and the output of the last encoder layer is the latent embedding ${\boldsymbol{Z}}^{(L)}={\left({\boldsymbol{C}}^{\prime },{\boldsymbol{S}}^{\prime}\right)}^{\mathrm{T}}$ whose ${\boldsymbol{C}}^{\prime }$ is the cell embedding and ${\boldsymbol{S}}^{\prime }$ is the spot embedding. ${\boldsymbol{Z}}^{(L)}$ is set as the input of the decoder. The decoder is symmetric to the encoder with the same number of layers, and the process of each decoder layer is the reversal of its symmetric encoder layer. There are normalized attention coefficients ${\overline{\alpha}}_{ij}^{(l)}$ in the $l$-th decoder (where $l=L-1,L-2,\dots, 1$) as ${\alpha}_{ij}^{(l)}$ in the $l$-th encoder. The $\left(l-1\right)$-th decoder layer is reconstructed from the $l$-th decoder layer by Formula (4).


(4) \begin{eqnarray*} {\overline{\boldsymbol{z}}}_i^{\left(l-1\right)}=\sum_{j\in{\boldsymbol{N}}_i}{\overline{\alpha}}_{ij}^{(l)}\sigma \left({\overline{\boldsymbol{W}}}^{(l)}{\overline{\boldsymbol{z}}}_j^{(l)}\right) \end{eqnarray*}

Here, ${\overline{\boldsymbol{z}}}_i^{(l)}$ denotes the node $i$ in the $l$-th decoder layer. After applying all decoder layers, the outputs of the last decoder layer ${\overline{\boldsymbol{z}}}_i^{(0)}$are the reconstructed profiles of node $i$. The autoencoder is trained by minimizing the reconstruction loss as shown in Formula (5) with Adam Optimizer.


(5) \begin{eqnarray*} \sum_{i=1}^{m+n}{\left\Vert{\boldsymbol{z}}_i^{(0)}-{\overline{\boldsymbol{z}}}_i^{(0)}\right\Vert}_2 \end{eqnarray*}

### Cell-to-spot mapping algorithm

To learn the cell-to-spot mapping matrix $\boldsymbol{M}$, each of whose elements represents the mapping score from a cell to a spot, we construct an objective function and try to minimize the objective function $f\left(\boldsymbol{M}\right)$ shown in Formula (6), which satisfies $\sum_{j=1}^n{M}_{ij}=1$ and $0\le{M}_{ij}\le 1$ by applying the softmax function along all spots for each cell.


(6) \begin{eqnarray*} f\left(\boldsymbol{M}\right)=&\, {\lambda}_d{\left\Vert \boldsymbol{m}-\boldsymbol{d}\right\Vert}_2-{\lambda}_g\nonumber \\ & \times \left(\sum_{k=1}^{d^{(L)}}\cos \left({\left({\boldsymbol{M}}^{\mathrm{T}}{\boldsymbol{C}}^{\prime}\right)}_{\ast, k},{\boldsymbol{S}}_{\ast, k}^{\prime}\right)+\sum_{j=1}^n\cos \left({\left({\boldsymbol{M}}^{\mathrm{T}}{\boldsymbol{C}}^{\prime}\right)}_{j,\ast },{\boldsymbol{S}}_{j,\ast}^{\prime}\right)\right)\nonumber \\ & +\alpha \sum_{i,j}\mathrm{KL}\left({\left({\boldsymbol{M}}^{\mathbf{T}}\boldsymbol{C}\right)}_{i,\ast },{\boldsymbol{S}}_{j,\ast}\right)-\frac{\lambda_1}{n}\left(\left(1-{\lambda}_2\right)\sum_{j=1}^n{\left\Vert{\left({\boldsymbol{M}}^{\mathrm{T}}{\boldsymbol{C}}^{\prime}\right)}_{j,\ast } \right. } \right. \nonumber \\ & \left. { \left.-{\boldsymbol{S}}_{\boldsymbol{j},\ast}^{\prime}\right\Vert}_2\!+\!{\lambda}_2\sum_{j=1}^n\sqrt{\left({\left({\boldsymbol{M}}^T{\boldsymbol{C}}^{\prime}\right)}_{j,\ast }\!-\!{\boldsymbol{S}}_{\boldsymbol{j},\ast}^{\prime}\right){\boldsymbol{\varSigma}}^{-1}{\left({\left({\boldsymbol{M}}^T{\boldsymbol{C}}^{\prime}\right)}_{j,\ast }\!-\!{\boldsymbol{S}}_{\boldsymbol{j},\ast}^{\prime}\right)}^{\mathrm{T}}}\right) \end{eqnarray*}

where ${\left(\cdotp \right)}_{j,\ast }$ and ${\left(\cdotp \right)}_{\ast, k}$ represent the vector composed of the $j$-th row and the vector of the $k$-th column in a matrix (·), respectively, and ${\lambda}_d,{\lambda}_g,\alpha, {\lambda}_1$, and ${\lambda}_2$ are five adjustable parameters.

The objective function $f\left(\boldsymbol{M}\right)$ consists of four terms. The first term is the density term (DeT): we enforce that the learned density distribution$\boldsymbol{m}$ for spots is as close as possible to the expected distribution $\boldsymbol{d}$. Each component ${m}_j$ of the vector $\boldsymbol{m}={\left({m}_1,\dots, {m}_n\right)}^{\mathrm{T}}$ is calculated using ${m}_j=\sum_{i=1}^m{M}_{ij}/m$ and regarded as the predicted cell density. The second term is the embedding-based cosine similarity term (CST) composed of two parts: the first part enforces that each column ${\left({\boldsymbol{M}}^{\mathrm{T}}{\boldsymbol{C}}^{\prime}\right)}_{\ast, k}$of reconstructed spatial embedding, which is predicted by the mapping matrix $\boldsymbol{M}$ and the cell embedding ${\boldsymbol{C}}^{\prime }$, is proportional to the column ${\boldsymbol{S}}_{\ast, k}^{\prime }$ of the spot embedding ${\boldsymbol{S}}^{\prime }$; the second part enforces that each row ${\left({\boldsymbol{M}}^{\mathrm{T}}{\boldsymbol{C}}^{\prime}\right)}_{j,\ast }$ is proportional to the row ${\boldsymbol{S}}_{j,\ast}^{\prime }$. The third term is the expressed-based MT: it enforces that the difference, represented by Kullback-Leibler divergence, between the merged gene expression of the mapped cells mapped to a spot and the expression of the spot should be minimized as much as possible. The last term is the embedding-based DiT composed of two distance metrics: the first part is the sum of the Euclidean distance between reconstructed spatial embedding ${\left({\boldsymbol{M}}^{\mathrm{T}}{\boldsymbol{C}}^{\prime}\right)}_{j,\ast }$ and spot embedding ${\boldsymbol{S}}_{j,\ast}^{\prime }$ for all spots $j=1,\dots, n$; the second part is the sum of the Mahalanobis distance between ${\left({\boldsymbol{M}}^{\mathrm{T}}{\boldsymbol{C}}^{\prime}\right)}_{j,\ast }$ and ${\boldsymbol{S}}_{j,\ast}^{\prime }$ for all spots $j=1,\dots, n$, in which${\boldsymbol{\varSigma}}^{-1}$ is the inverse matrix of the covariance matrix $\boldsymbol{\varSigma} = Cov\left({\boldsymbol{M}}^T{\boldsymbol{C}}^{\prime },{\boldsymbol{S}}^{\prime}\right)$. Gradient-based optimization with the PyTorch library is employed to minimize the objective function $f\left(\boldsymbol{M}\right)$.

Once the matrix$\boldsymbol{M}$ is obtained, for each cell $i$, the spot $j$ with the highest probability value ${M}_{ij}$ among all spots will be labeled. Then, for each spot $j$, if the number of labeled cells is less than the estimated number of cells ${s}_j$, all these labeled cells are assigned to the spot $j$, otherwise, the top ${s}_j$ labeled cells with the highest values are assigned to spot $j$. Subsequently, the assigned cells and the spots that have been filled to the full are removed from $\boldsymbol{M}$. The above process is repeated until there are no more spots or cells left.

### Benchmark datasets and case study for Cell2Map evaluation

To fairly evaluate the performance of Cell2Map, we first utilized simulated SRT datasets. The simulated SRT datasets were designed in CytoSPACE [27] and created from the mouse cerebellum and hippocampus data generated using Slide-seq [5], a platform with spatial single-cell resolution and limited transcriptome representation. The single-cell expression profiles in the Slide-seq data were first replaced by the most correlated profiles from an independent scRNA-seq dataset [34] of the same brain region. Then, the spatial space was split into grids and the replaced expression profiles in each grid were merged together into pseudo-bulk transcriptomes to form a spot. To simulate SRT data with different resolutions, the grid size was adjusted so that each spot covers an average of 5, 15, and 30 cells. Finally, to simulate the differentiation of technology and platform between scRNA-seq and SRT datasets, varying amounts of noise (noise perturbation levels of 5%, 10%, and 25%) were added to the scRNA-seq dataset. The detailed information of simulated benchmark datasets is summarized in Supplementary Table S1.

To evaluate performance on real SRT datasets, we next ran Cell2Map on data of mouse MI and two types of human cancer: human ductal carcinoma *in situ* (DCIS) and breast cancer (summarized in Supplementary Table S2). The scRNA-seq data in the MI dataset consists of 11 492 single cardiac cells from a mouse model of pressure overload [35], and the MI SRT data were sequenced from some frozen samples of 7-day post-MI [36]. The DCIS dataset consists of two samples, a pure DCIS sample (DCIS1) and a DCIS tissue with synchronous invasive ductal carcinoma (DCIS2), both obtained from CellTrek [26]. The DCIS1 sample consists of 6828 single cells and 1567 SRT spots. The DCIS2 sample consists of 3748 single cells and 2063 SRT spots. The breast cancer dataset consists of a HER2^+^ FFPE (formalin-fixed, paraffin-embedded) breast cancer scRNA-seq atlas [37] and a fresh frozen breast cancer specimens profiled by the Visium platform of 10X Genomics.

### Parameter training and sensitivity analysis

The weights of GATE in Cell2Map were optimized using the Adam optimizer implemented in PyTorch. The initial learning rate was set to 0.1, with a default batch size of 100 and 200 training epochs. The Cell2Map objective function contains four major objective terms controlled by five weighting hyperparameters. The default hyperparameters were selected by grid search using the simulated mouse cerebellum and mouse hippocampus benchmark datasets with 5% noise (Supplementary Tables S3 and S4). The searched ranges were ${\lambda}_d\in \left\{1,2,4,8,16\right\}$, $\alpha \in \left\{1,3,5,7,9\right\},{\lambda}_1\in \left\{\mathrm{0.001,0.01},\mathrm{0.05,0.1,1}\right\}$, and ${\lambda}_2\in \left\{\mathrm{0.3,0.5,0.7,0.9}\right\}$. Based on this grid search, the default values were set to ${\lambda}_g=1$, ${\lambda}_d=8$, $\alpha =7$, ${\lambda}_1=0.01$, and ${\lambda}_2=0.7$.

To assess parameter robustness, we performed sensitivity analyses on the simulated benchmark datasets. First, ${\lambda}_1$ and ${\lambda}_2$ were varied while fixing ${\lambda}_g=1$, ${\lambda}_d=8$, $\alpha =7$. As shown in Supplementary Table S3, the default setting ${\lambda}_1=0.01$ and ${\lambda}_2=0.7$ achieved the best overall performance across the tested mouse cerebellum and mouse hippocampus datasets under different spot-density conditions. We then varied ${\lambda}_d$ and $\alpha$ while fixing ${\lambda}_1=0.01$ and ${\lambda}_2=0.7$. As summarized in Supplementary Table S4, the default setting ${\lambda}_d=8$and $\alpha =7$provided the most stable and accurate performance across the benchmark datasets. The dataset-specific values of ${\lambda}_1$ and ${\lambda}_2$ used for real-data analyses are listed in Supplementary Table S5. We further evaluated the sensitivity of Cell2Map to graph-construction and training parameters. The KNN setting of $k=6$ achieved the best performance among the tested values, with nearby values showing comparable results (Supplementary Table S6). Meanwhile, the spatial neighbor radius of 5 achieved the best performance among the tested radii (Supplementary Table S7). Similarly, the default learning rate of 0.1 yielded the best performance among the tested learning rates (Supplementary Table S8).

### Methods comparison and evaluation metrics

We compared Cell2Map with three popular cell-to-spot alignment algorithms: Tangram [25], CytoSPACE [27], and Celloc [28]. Tangram is the first widely used single-cell-to-spot mapping algorithm. CytoSPACE and Celloc are the two best performing mapping methods on the benchmark data in the latest study. We excluded CellTrek [26] because it can assign the same cell to multiple spots. We used default settings for all methods. All experiments were run on a single machine with an NVIDIA RTX 4090 GPU (24 GB), an Intel Core i9-13900K CPU, and 64 GB RAM. We employed two metrics to evaluate the performance of each method on the benchmark data for the single-cell-to-spot mapping tasks. The first metric is the single-cell mapping precision ($P{r}_{\mathrm{C}2\mathrm{S}}$) defined as Formula (7), representing the percentage of cells mapped to the correct spots in a cell type.


(7) \begin{eqnarray*} {\mathit{\Pr}}_{\mathrm{c}2\mathrm{s}}=\frac{T{P}_{\mathrm{c}2\mathrm{s}}}{N_{\mathrm{c}2\mathrm{s}}} \end{eqnarray*}

In Formula (7), $T{P}_{\mathrm{c}2\mathrm{s}}$ denotes the number of cells that were assigned to correct spots, and ${N}_{\mathrm{c}2\mathrm{s}}$ denotes the number of all mapped cells with known ground truth spots for a cell type. The second is the overall accuracy metric ($ACC$), which calculates the accuracy of all cells (from all cell types) mapped to spots, defined as


(8) \begin{eqnarray*} ACC=\frac{N_{acc}}{N_{total}} \end{eqnarray*}

where ${N}_{acc}$ represents the number of cells assigned correctly, and ${N}_{total}$ is the number of all assigned cells for all cell types.

To evaluate the mapping accuracy of a certain cell type in real data, the overlapping rate between mapped spot areas and annotated spot area for a certain cell type is defined as


(9) \begin{eqnarray*} {R}_{overlap}=\frac{S_{map}\cap{S}_{annotated}}{S_{map}\cup{S}_{annotated}} \end{eqnarray*}

where ${S}_{map}$ refers to the number of spots fully mapped by this cell type and ${S}_{annotated}$stands for the number of spots fully annotated as this cell type.

### Detect differentially expressed genes and data visualization

Differentially expressed genes (DEGs) were identified to characterize transcriptional differences between the selected cell populations versus others by using Wilcoxon rank-sum test in the SCANPY (version1.9.6) [31]. To control for multiple hypothesis testing, *p*-values were adjusted using the Benjamini-Hochberg false discovery rate. Genes satisfying the predefined thresholds of adjusted *P*-value <.05 and log_2_(fold change) >0.5 were considered significant DEGs. To visualize the DEG results, volcano plots were generated by plotting log_2_(fold change) against the $-$log_10_(adjusted *p*-value). Heatmaps were further generated to display the spatial expression patterns of representative DEGs across cells. To support biological interpretation, the functional annotations of significant DEGs were obtained from the GeneCards database [38]. In addition, the relevance of selected marker genes to breast cancer was manually curated through literature review using PubMed, focusing on their reported roles in tumor initiation, progression, metastasis, therapeutic response, and tumor microenvironment regulation. Uniform manifold approximation and projection (UMAP) was used to project high-dimensional data into a two-dimensional space for visualization of cell populations and clustering patterns [39].

## Results

### Cell2Map exhibits superior performance on benchmark datasets

**Figure 2:**
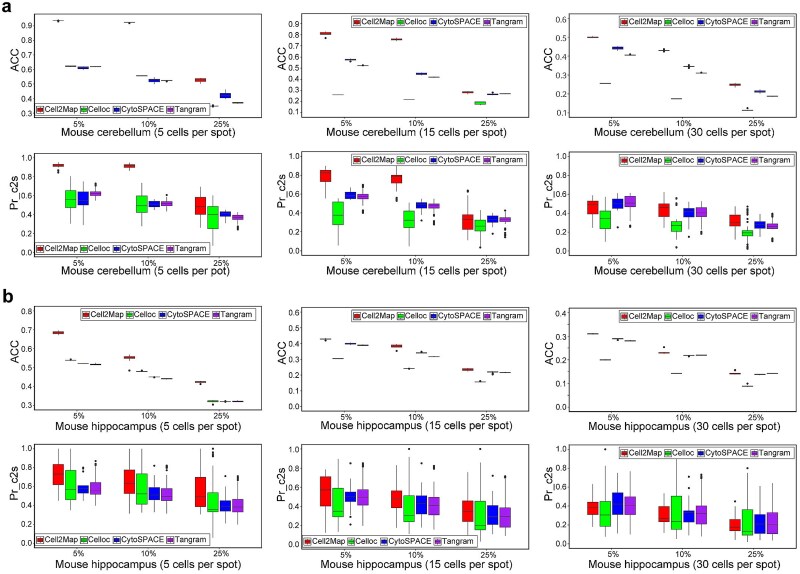
Performance comparison among four methods; the ACC and $P{r}_{\mathrm{C}2\mathrm{S}}$ scores of four cell-to-spot alignment algorithms on each cell type of the simulated mouse cerebellum (a) and mouse hippocampus (b) datasets with different noise levels (5%, 10%, and 25%); the mean and SDs of ACC scores are presented in Supplementary Tables S9 and S10, and $P{r}_{\mathrm{C}2\mathrm{S}}$ scores are presented in Supplementary Tables S11 and S12.

We evaluated the performance of Cell2Map using simulated spatial transcriptomics datasets derived from mouse cerebellum and hippocampus scRNA-seq data. The simulated cerebellum datasets contain 11 cell types, while the hippocampus datasets contain 17 cell types. For each dataset, we assessed single-cell mapping precision ($P{r}_{C2S}$) for individual cell types and ACC across all cell types. To ensure robustness, each method was run 10 independent times using recommended parameter settings. We evaluated performance under varying experimental conditions, including three noise levels (5%, 10%, and 25%) and three spot resolutions corresponding to an average of 5, 15, and 30 cells per spot. We compared Cell2Map against three state-of-the-art cell-to-spot alignment methods, Celloc, CytoSPACE, and Tangram. The distributions of ACC and $P{r}_{C2S}$ scores are shown as boxplots in Fig. 2. The detailed ACC scores are summarized in Supplementary Tables S9 and S10, and the $P{r}_{C2S}$ scores for the 11 cell types in the mouse cerebellum dataset and the 17 cell types in the mouse hippocampus dataset are presented in Supplementary Tables S11 and S12, respectively.

As shown in Fig. 2a and b, when the spot resolution is high (average of five cells per spot), Cell2Map consistently and significantly outperforms the other three methods on both mouse cerebellum and hippocampus datasets across all tested noise levels (5%, 10%, and 25%). The performance gains are evident across nearly all cell types, demonstrating Cell2Map’s superior ability to resolve fine-grained cellular localization under low-density conditions. For datasets with an intermediate resolution (15 cells per spot), Cell2Map maintains a clear advantage at low and moderate noise levels (5% and 10%), while performance differences among methods diminish at 25% noise, where increased spot complexity and noise limit discrimination power for all approaches. At lower-resolution settings (30 cells per spot), the performance gap between methods further narrows across noise levels. Nevertheless, Cell2Map consistently achieves equal or slightly higher $P{r}_{C2S}$ scores compared with competing methods, indicating stable performance even in highly mixed spot scenarios.

For the simulated mouse hippocampus datasets spanning different noise levels and cell densities, we further evaluated the overall ACC scores across all cell types. Because the ACC values showed minimal variation among the 10 repeated experiments for each dataset, we summarized the results using the mean ACC score and corresponding SD for each dataset (Supplementary Table S10). Notably, the SDs of ACC scores for Cell2Map across all datasets were consistently below 0.02, indicating stable performance across repeated runs. Across all simulated datasets, Cell2Map consistently achieved higher ACC scores than the other three methods.

We also evaluated the computational efficiency of Cell2Map by comparing runtime and peak GPU memory usage with Celloc, CytoSPACE, and Tangram on representative simulated and real datasets. As shown in Supplementary Table S13, Cell2Map completed the simulated cerebellum and hippocampus analyses within ~4–5 min and completed the real-data analyses within ~2 min. In addition, Cell2Map showed comparable peak graphics processing unit (GPU) memory usage relative to the other methods (Supplementary Table S14). Although Cell2Map required slightly higher GPU memory for some simulated datasets, its memory usage remained within the capacity of a standard high-end GPU and was comparable across real biological datasets. Together, these results demonstrate that Cell2Map provides more accurate and robust cell-to-spot mapping across diverse simulation settings, particularly under low cell-density and high-noise conditions, while maintaining practical runtime and memory requirements as spot complexity increases.

### Multiple terms in the Cell2Map objective function and the GATE model interactively improve the performance

Cell2Map employs a multi-term objective function that integrates four complementary components: a cell DeT, an embedding-based CST, an embedding-based DiT, and an expression-based MT. To quantify the individual contribution of each term and assess their interactions, we conducted systematic ablation experiments by removing one term at a time from the full objective function. As summarized in Table 1, removing any component leads to a noticeable degradation in performance, confirming that all four terms contribute meaningfully to accurate cell-to-spot mapping.

**Table 1:** The results of ablation experiments on the simulated cerebellum dataset with 5% noise and five-cell-per-spot resolution.

Objective function		All terms	Without DeT	Without CST	Without DiT	Without MT
ACC		**0.9356** (0.002)	0.8833 (0.004)	0.9314 (0.001)	0.8906 (0.014)	0.0009 (0.000)
$P{r}_{c2s}$ of 11 cell types	Astrocyte	**0.9296** (0.003)	0.8319 (0.007)	0.9244 (0.001)	0.8207 (0.019)	0 (0.000)
	Bergmann	**0.9286** (0.001)	0.8529 (0.004)	0.9146 (0.001)	0.8482 (0.015)	0.0007 (0.000)
	Choroid	**0.8634** (0.007)	0.8407 (0.008)	0.8581 (0.002)	0.8344 (0.014)	0 (0.000)
	Endothelial	**0.9221** (0.001)	0.8188 (0.006)	0.9122 (0.001)	0.9058 (0.011)	0.0017 (0.002)
	Fibroblast	**0.9078** (0.003)	0.8601 (0.001)	0.9047 (0.000)	0.8656 (0.018)	0 (0.000)
	Granule	**0.9525** (0.002)	0.9028 (0.002)	0.9509 (0.000)	0.9167 (0.014)	0.0008 (0.000)
	Microglia	**0.9485** (0.001)	0.8861 (0.006)	0.9418 (0.001)	0.9165 (0.017)	0 (0.000)
	Nnat	**0.9093** (0.003)	0.8689 (0.003)	0.9025 (0.001)	0.8416 (0.017)	0 (0.000)
	Oligodendrocyte	**0.9177** (0.004)	0.9071 (0.001)	0.9142 (0.001)	0.8722 (0.010)	0.0013 (0.000)
	Purkinje	**0.9075** (0.001)	0.8974 (0.006)	0.8945 (0.000)	0.8599 (0.017)	0.0011 (0.000)
	Pvalb	**0.9296** (0.003)	0.8423 (0.010)	0.9217 (0.000)	0.8869 (0.009)	0 (0.000)

Bold values indicate the highest value for each evaluation metric among the compared methods.

In particular, removing the MT results in a near-zero overall accuracy and cell-to-spot precision across all cell types, indicating that this term is essential for establishing meaningful correspondence between scRNA-seq cells and spatial spots. MT is the only term in the objective function that establishes a direct correspondence between scRNA-seq cells and SRT spots in the original gene-expression space, and the remaining three terms (DeT, CST, DiT) act as regularization or refinement terms operating in the latent embedding space or on cell-density distributions. Without MT, the optimization has no anchor that links cell identity to spot identity through gene expression, and the model fails to recover biologically meaningful mappings. The DeT also plays a critical role by enforcing consistency between predicted and observed cell densities across spots. Its removal leads to substantial decreases in overall accuracy (ACC drops from 0.9356 to 0.8833) and pronounced performance loss across most cell types, highlighting its importance in maintaining realistic spatial cell allocations. The DiT further enhances performance by encouraging mapped cells and spots to be close in latent space. Excluding this term results in consistent accuracy reductions (ACC = 0.8906), demonstrating that distance-based constraints provide complementary structural information beyond expression alignment alone. Finally, the CST contributes additional robustness by promoting directional consistency between cell and spot embeddings. Although its removal causes the smallest performance decline among the four terms (ACC = 0.9314), the consistent decrease across multiple cell types indicates that CST still provides a meaningful, albeit auxiliary, contribution to overall mapping accuracy.

The spatial neighbor graph in Cell2Map is constructed solely from the spatial coordinates of SRT spots. It encodes the intrinsic spatial geometry of the tissue section, where neighboring spots are physically adjacent in the SRT data. To evaluate the contribution of the graph-based representation, we replaced the GATE architecture with either principal component analysis (PCA) or a conventional autoencoder to obtain low-dimensional latent embeddings, while keeping the same four-term objective function. As shown in Table 2 and Supplementary Table S15, both PCA-based and autoencoder-based baselines achieved lower ACC and $P{r}_{c2s}$ scores than the full Cell2Map model. For example, on the simulated mouse cerebellum dataset with 5% noise and five-cell-per-spot resolution, Cell2Map achieved an ACC of 0.9356, compared with 0.7216 for PCA plus the four-term objective and 0.8505 for autoencoder plus the four-term objective. Similarly, on the simulated mouse hippocampus dataset, Cell2Map achieved an ACC of 0.6848, compared with 0.5656 for PCA plus the four-term objective and 0.5082 for autoencoder plus the four-term objective. These results demonstrate that the graph-based GATE architecture provides a clear performance advantage and that the improvement is not simply due to the four-term objective function alone. Together, these ablation results demonstrate that the graph-based GATE architecture provides a clear performance advantage beyond the four-term objective function alone, while the four objective terms act synergistically to achieve accurate, stable, and biologically consistent cell-to-spot assignments under noisy and heterogeneous conditions.

**Table 2:** The results of ablation experiments on the simulated mouse cerebellum dataset with 5% noise and five-cell-per-spot resolution with two different baselines.

Model		PCA+ four-term objective	Autoencoder + four-term objective	Cell2Map
$ACC$		0.7216	0.8505	**0.9356**
$P{r}_{c2s}$ of 11 cell types	Astrocyte	0.7040	0.8064	**0.9296**
	Bergmann	0.6881	0.7782	**0.9286**
	Choroid	0.6661	0.8314	**0.8634**
	Endothelial	0.7153	0.8253	**0.9221**
	Fibroblast	0.7740	0.8269	**0.9078**
	Granule	0.7469	0.8872	**0.9525**
	Microglia	0.7770	0.8781	**0.9485**
	Nnat	0.6429	0.8459	**0.9093**
	Oligodendrocyte	0.7543	0.8222	**0.9177**
	Purkinje	0.6494	0.8292	**0.9075**
	Pvalb	0.7079	0.8318	**0.9296**

Bold values indicate the highest value for each evaluation metric among the compared methods.

### Cell2Map identifies the tumor heterogeneity of DCIS

**Figure 3:**
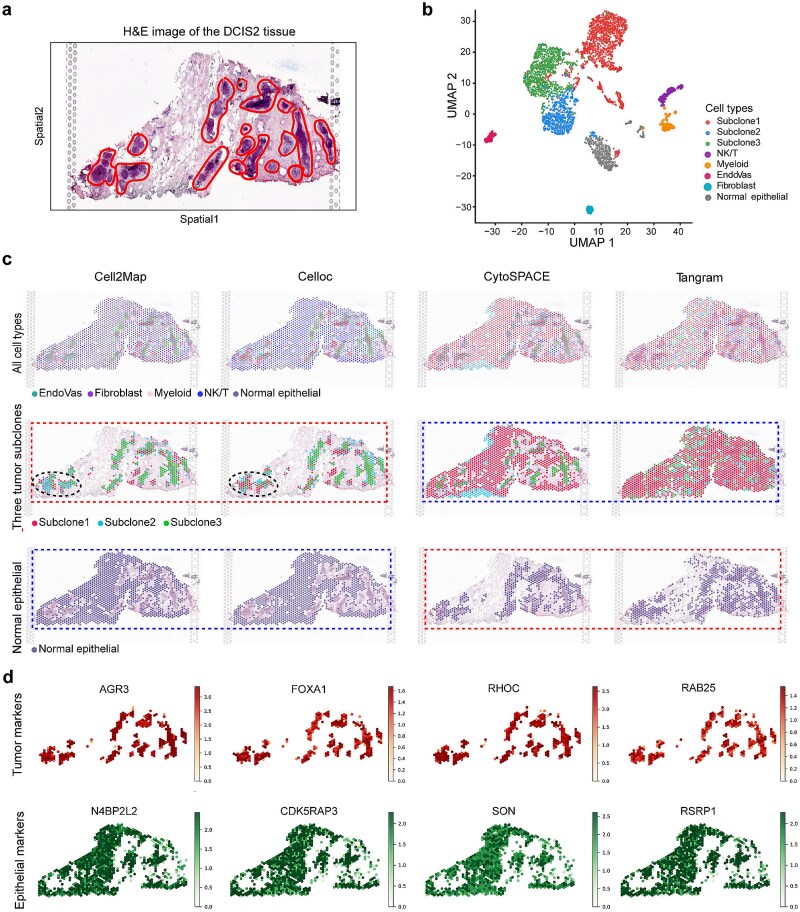
Visualization of mapping DCIS2 scRNA-seq data to DCIS2 SRT data; (a) the H&E image in the DCIS2 SRT data with histologically annotated ductal tumor regions outlined; (b) UMAP plot of DCIS2 scRNA-seq data 3587 cells annotated with major cell types, including three tumor subclones; (c) spatial mapping results of scRNA-seq onto SRT spots; dashed outlines indicate regions of correct localization and/or mis-localization relative to histologically defined tumor areas, illustrating differences in spatial specificity and tumor-normal separation across methods; (d) spatial expression of markers for tumor and epithelial cells.

Tumor cells generally consist of multiple subclones, and it is difficult to distinguish them directly through SRT data and H&E images. To validate the ability of Cell2Map to distinguish tumor cell subtypes, here we used the DCIS2 dataset of ductal carcinoma and mapped the scRNA-seq cells to the SRT spots. The scRNA-seq data in the DCIS2 dataset have been annotated as four nontumor cell types [EndoVas, Fibroblast (FB), Myeloid, and natural killer T cells (NK/T)] and three subclones of tumor cells (Subclone1, Subclone2, and Subclone3) [26]. On the tissue H&E image of the DCIS2 SRT data, ductal tumor regions are enclosed with red lines (Fig. 3a). In the DCIS2 scRNA-seq data, the tumor cells can be clustered into three clusters, corresponding to three subclones, respectively (Fig. 3b). We assume that the average number of cells per spot is five. After running Cell2Map and the compared algorithms separately on DCIS2 dataset, we obtained the mapping relationship from scRNA-seq cells to SRT spots. Thus, these scRNA seq cells labeled with cell types were spatially reconstructed.

As shown in Fig. 3c, spatial reconstructions of all cell types defined in the scRNA-seq reference are visualized across tissue spots, with distinct colors indicating different cell populations. Overall, Cell2Map, Celloc, CytoSPACE, and Tangram produce qualitatively different spatial distributions, revealing marked differences in their ability to resolve tumor architecture and cellular heterogeneity. Cell2Map and Celloc both localize tumor cells (annotated as three tumor subclones) to regions that are highly consistent with the tumor areas identified from the H&E image in Fig. 3a, indicating accurate tumor localization by both methods. However, Cell2Map produces a more structured and biologically coherent spatial organization of tumor subclones. Specifically, subclone 1 is predominantly concentrated on the left side of the tissue section, subclone 3 is mainly distributed in the middle and right regions, and subclone 2 is preferentially localized along tumor boundaries (e.g. regions highlighted by black dotted circles). In contrast, Celloc shows less spatial separation among the three subclones, with greater intermixing across tumor regions. By comparison, CytoSPACE and Tangram display markedly reduced spatial specificity for tumor cells. We also computed the overlapping rate ${R}_{overlap}$ between the mapped spot areas and the annotated spot area for the three tumor subclones for each compared method, and found that Cell2Map achieves the highest overlapping rate (${R}_{overlap}=0.7863$) (Supplementary Table S16). Tumor subclones predicted by these methods are highly colocalized with normal epithelial cells (blue dotted triangles in Fig. 3c), suggesting substantial misassignment of malignant and non-malignant populations. Importantly, Cell2Map clearly separates normal epithelial cells from tumor regions, as illustrated in the third-row subpanels of Fig. 3c, where normal epithelial cells are largely excluded from malignant areas. In contrast, CytoSPACE and Tangram incorrectly place a substantial fraction of normal epithelial cells within tumor regions (red dotted triangles in Fig. 3c), leading to pronounced overlap with malignant cell distributions.

To further validate the spatial assignments generated by Cell2Map, we identified DEGs between tumor cells and normal epithelial cells based on the mapped scRNA-seq data (Supplementary Fig. S1). The volcano plots highlight genes significantly enriched in tumor cells versus other cell types (Supplementary Fig. S1a) and genes preferentially expressed in normal epithelial cells (Supplementary Fig. S1b). Several well-known tumor-associated markers, including RAB25, FOXA1, CD24, AGR2, FXYD3, AGR3, CLDN4, and RHOC, show strong upregulation in tumor cells [40–42], whereas genes such as N4BP2L2, SON, CDK5RAP3, DDX17, RSRP1, PNISR, CCNL2, and ANKRD30A are significantly enriched in epithelial cells [43–45]. These DEG results provide candidate markers for validating the spatial reconstruction of tumor and epithelial populations (see annotation details in Supplementary Table S17). We next examined the spatial expression patterns of representative markers selected from these DEG analyses (upper row in Fig. 3d and Supplementary Fig. S1c). Tumor-associated markers exhibit strong expression signals that are predominantly localized within the ductal tumor regions identified in the H&E image (Fig. 3a). Meanwhile, markers associated with normal epithelial cells display expression patterns largely outside tumor boundaries and are consistent with non-malignant epithelial regions in the tissue section (lower row in Fig. 3d and Supplementary Fig. S1c). Notably, the clear spatial separation between tumor marker expression and normal epithelial marker expression provides independent biological evidence and demonstrates that Cell2Map effectively distinguishes malignant from non-malignant epithelial populations.

### Cell2Map detects spatial tumor microenvironment in diverse cancer types

**Figure 4:**
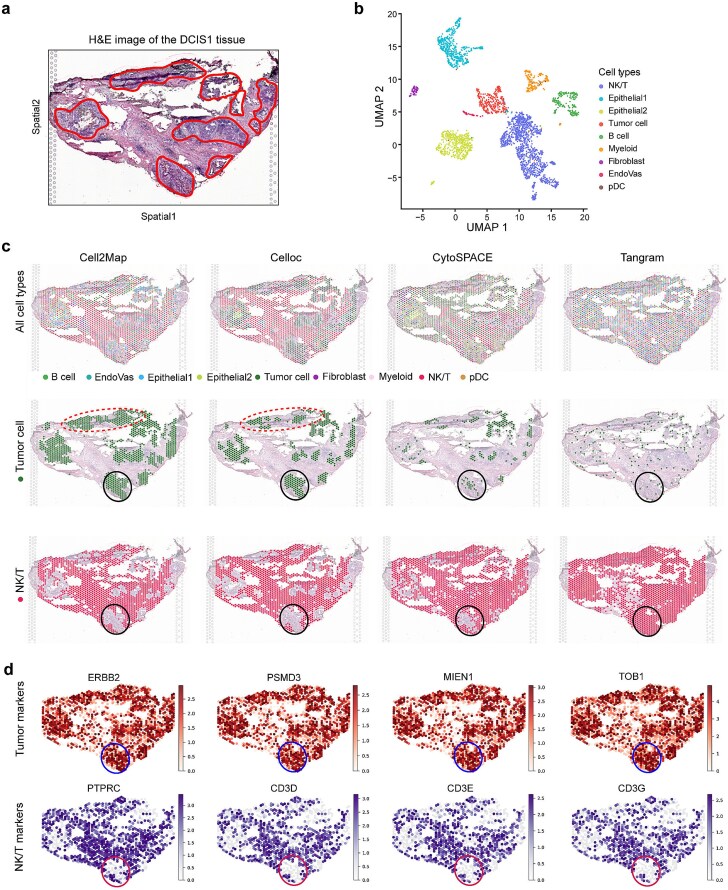
Spatial reconstruction of the DCIS1 tumor microenvironment; (a) H&E image of the DCIS1 tissue section from the SRT data with annotated ductal tumor regions surrounded; (b) UMAP plot of the matched DCIS1 scRNA-seq data annotated with major cell types; (c) spatial mapping results of scRNA-seq cell types onto SRT spots using four methods; dashed outlines mark representative regions where methods differ in tumor coverage (dashed ellipse) and in tumor cores (circle), illustrating differences in spatial specificity and false-positive assignments across methods; (d) spatial expression of representative markers for tumor cells (upper row: ERBB2, PSMD3, MIEN1, TOB1) and NK/T cells (lower row: PTPRC, CD3D, CD3E, CD3G); circled regions indicate the histologically annotated DCIS tumor boundary; color scales are normalized gene expression levels and are shown consistently across subpanels.

To evaluate Cell2Map’s ability to reconstruct the spatial tumor microenvironment, we applied Cell2Map, Celloc, CytoSPACE, and Tangram to the DCIS1 dataset. The H&E image of the tissue section includes histologically annotated DCIS regions (Fig. 4a), and the matched scRNA-seq reference resolves into nine major cell types (Fig. 4b). As shown in Fig. 4c, Cell2Map provides the most accurate spatial reconstruction by mapping tumor cells predominantly to the histologically defined DCIS regions, with ${R}_{overlap}=0.7656$ (Supplementary Table S16), while localizing NK/T cells to the peritumoral and stromal areas surrounding the ducts. In contrast, although Celloc also assigns tumor cells within the DCIS-labeled regions, the predicted tumor regions are noticeably smaller than the annotated tumor areas (e.g. red dashed circles in Fig. 4c). Importantly, Cell2Map shows reduced mislabeling of immune cells within tumor cores. In the NK/T cell maps (third row of Fig. 4c), Cell2Map exhibits lower spurious localization of NK/T cells inside DCIS tumor regions (black circles), whereas other methods display more scattered NK/T assignments within tumor interiors. This pattern suggests that Cell2Map better preserves the expected spatial segregation between malignant compartments and immune-rich peripheral regions, thereby yielding a more faithful reconstruction of tumor microenvironment structure with fewer false-positive assignments.

We next examined DEGs and their spatial expression patterns for tumor and immune cell populations (Supplementary Fig. S2 and Fig. 4d). DEG analysis identified several genes significantly enriched in tumor cells compared with other cell types, including ERBB2, PSMD3, MIEN1, TOB1, PHB1, MRPL27, COPRS, and HOXB2 [46–48], while NK/T cells showed strong enrichment of immune lineage markers such as PTPRC, TRAC, TRBC1, TRBC2, CD3D, CD3E, CD3G, and CD2 [49–51] (Supplementary Fig. S2a and b). These genes are widely recognized as breast cancer marker genes and their coordinated upregulation is consistent with malignant epithelial identity and supports their functional relevance to breast cancer development and progression (see annotation details in Supplementary Table S18). Mapping the spatial expression of representative markers further confirmed the accuracy of the reconstructed tissue architecture. As shown in Fig. 4d, tumor-associated markers such as ERBB2, PSMD3, MIEN1, and TOB1 display strong expression signals that are predominantly localized within the DCIS tumor regions identified in the H&E image (Fig. 4a), consistent with the spatial tumor localization predicted by Cell2Map. The annotated tumor boundaries (circles in Fig. 4d) further highlight the spatial concordance between marker expression and histologically defined malignant regions. In contrast, immune markers including PTPRC, CD3D, CD3E, and CD3G are enriched in regions surrounding the tumor ducts rather than within the tumor cores, consistent with the spatial distribution of NK/T cells observed in the reconstructed maps (Fig. 4c). Additional markers highlighted in Supplementary Fig. S2c further support this spatial segregation, with tumor markers (PHB1, MRPL27, COPRS, HOXB2) and NK/T markers (CD2, TRAC, TRBC1, TRBC2) exhibiting spatial patterns that align with the tumor and immune compartments inferred by Cell2Map.

We further evaluated spatial mapping performance in an MI dataset. The H&E image of the 10X Visium SRT data from a frozen sample at 7 days post-MI shows macrophages (MPs) diffusing outward from the fibrotic core (highlighted by red lines in Supplementary Fig. S3a). UMAP clustering of the scRNA-seq reference data identified five major cell types, including cardiomyocytes, endothelial cells, FBs, MPs, and smooth muscle cells (Supplementary Fig. S3b). Consistent with the biological expectation, Cell2Map accurately localized FBs and MPs within the fibrotic region and showed better agreement with known pathological patterns than Celloc, CytoSPACE, and Tangram (Supplementary Fig. S3c). In particular, CytoSPACE labeled very sparse FBs, indicating low sensitivity and potential under-detection. Tangram, while identifying more FBs, showed broader spatial distribution beyond the fibrotic region, suggesting increased false positives. These results demonstrate Cell2Map’s superior precision and biological consistency in mapping fibrosis-associated cell types in the MI spatial dataset.

### Cell2Map is robust in mapping different scRNA-seq datasets to breast cancer SRT data

**Figure 5:**
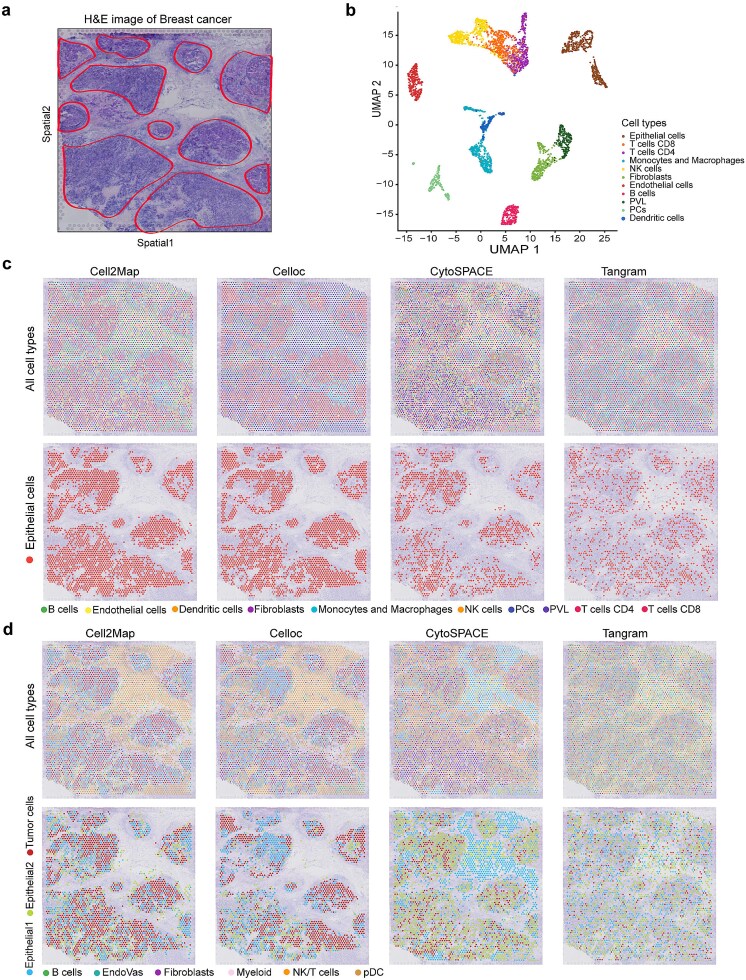
Robust cell-to-spot mapping of a breast cancer SRT dataset using different scRNA-seq reference datasets; (a) H&E-stained image of a 10X Visium breast cancer SRT tissue section, with histologically annotated tumor regions outlined; (b) UMAP plot of the scRNA-seq reference dataset annotated with major cell types; (c) spatial mapping results using a scRNA-seq reference in which epithelial cells are not subdivided into malignant and non-malignant subtypes (HER2+ FFPE breast cancer reference); (d) spatial mapping results using an alternative scRNA-seq reference DCIS1 that explicitly separates epithelial cells into tumor cells and two normal epithelial subtypes.

To assess the robustness of Cell2Map to different scRNA-seq reference datasets, we evaluated its performance using two independent references mapped to the same SRT sample. Specifically, we used (1) the DCIS1 scRNA-seq dataset analyzed previously and (2) a HER2+ FFPE breast cancer scRNA-seq dataset reported in CellTrek [26]. Each reference was independently mapped, using Cell2Map, Celloc, CytoSPACE, and Tangram, to a 10X Visium SRT dataset generated from a fresh-frozen invasive ductal carcinoma breast tissue section. Tumor regions were annotated based on the corresponding H&E image (Fig. 5a), and the average number of cells per spot was set to five for all methods.

The HER2+ FFPE scRNA-seq reference resolves 11 major cell types (Fig. 5b). When mapping this reference to the SRT data (Fig. 5c), Cell2Map localized epithelial cells more accurately to the histologically defined tumor regions than the other methods, with an overlap rate of ${R}_{overlap}=0.7592$ (Supplementary Table S16). Moreover, Cell2Map produced biologically plausible distributions of immune cells, particularly NK/T cells, around tumor boundaries and stromal regions. In contrast, CytoSPACE and Tangram yielded sparse and fragmented epithelial cell assignments, suggesting reduced sensitivity in capturing tumor-associated epithelial populations. We then examined differentially expressed tumor epithelial markers and their spatial expression patterns. Differential expression analysis identified several genes strongly enriched in epithelial populations, including AZGP1, WFDC2, TACSTD2, TM4SF1, EMP1, AK5, FXYD3, and TPM1 (Supplementary Fig. S4a, see annotation details Supplementary Table S19), which are frequently associated with epithelial and tumor-related processes [52–54]. Visualization of these markers across the spatial transcriptomics tissue section shows widespread expression signals consistent with epithelial-rich regions of the breast tissue, supporting the reliability of the epithelial cell assignments derived from Cell2Map (Supplementary Fig. S4b).

We further evaluated robustness using the DCIS1 scRNA-seq reference, which includes nine cell types and explicitly separates epithelial cells into tumor cells and two normal epithelial subtypes. As shown in Fig. 5d, Cell2Map consistently mapped tumor cells and epithelial subtypes to the annotated tumor regions with higher spatial coverage and coherence than competing methods. Although Celloc also placed epithelial and tumor cells within tumor regions, the number and spatial extent of mapped tumor cells were lower than those produced by Cell2Map. CytoSPACE and Tangram again exhibited sparse, diffuse, or inconsistent mappings, particularly for tumor-associated epithelial populations. We then examined the differential expression and spatial distribution of lineage-specific marker genes. Differential expression analysis identified several genes significantly enriched in NK/T cells, including CD3D, CD3E, PTPRC, IL32, CD2, CD3G, FYB1, and IL7R [49, 55, 56] (Supplementary Fig. S5a, see annotation details Supplementary Table S20). Visualization of their spatial expression patterns revealed that these immune markers are primarily localized in scattered stromal and peritumoral regions rather than within epithelial tumor cores (Supplementary Fig. S5b), consistent with the NK/T cell distributions reconstructed by Cell2Map. In parallel, tumor-associated markers identified from tumor-versus-other differential expression analysis, such as HOXB3, SIX1, SOX11, ONECUT2, EPN3, PRR15L, QPRT, and ST8SIA6-AS1 [57–59], display strong spatial enrichment in epithelial-rich tumor regions (Supplementary Fig. S6, see annotation details Supplementary Table S20). The spatial segregation between epithelial/tumor markers and immune NK/T markers further supports the accuracy of the reconstructed tumor microenvironment. Collectively, these results demonstrate that Cell2Map is robust to variation in scRNA-seq reference composition and annotation granularity, consistently reconstructing tumor regions and associated immune microenvironments with higher spatial fidelity than existing mapping approaches.

## Discussion and conclusion

In this study, we introduce Cell2Map, a deep learning framework for high-resolution mapping of scRNA-seq cells to SRT spots. By jointly modeling transcriptional similarity and spatial structure using a GATE and a multi-term objective function, Cell2Map addresses key challenges in cell-to-spot alignment, including data sparsity, noise, and heterogeneous spot composition. Systematic evaluations on simulated benchmarks and multiple real cancer datasets demonstrate that Cell2Map consistently outperforms existing methods in both single-cell mapping precision and overall accuracy. Our benchmark analyses show that Cell2Map achieves superior performance across a wide range of noise levels and spot resolutions. Notably, Cell2Map exhibits high sensitivity in detecting true cell-to-spot associations under low-density and noisy conditions, while maintaining stable performance as spot complexity increases. Ablation studies further confirm that the four objective terms, expression-based mapping, cell-density regularization, and embedding-based similarity and distance constraints, act synergistically to improve robustness and prevent overfitting, highlighting the importance of integrating complementary signals during optimization.

In real-world applications, Cell2Map more faithfully reconstructs tissue architecture and tumor microenvironments than competing approaches. Across multiple breast cancer datasets, including DCIS and invasive carcinoma samples, Cell2Map accurately localizes tumor cells to histologically annotated regions, resolves intratumoral subclone structure, and minimizes false-positive assignments of immune or normal epithelial cells within tumor cores. These results demonstrate Cell2Map’s ability to preserve biologically meaningful spatial boundaries and cell-type segregation, which is critical for downstream studies of tumor heterogeneity and immune interactions. Importantly, Cell2Map is robust to variation in scRNA-seq reference datasets, performing consistently across references with different cell-type compositions and annotation granularity. This flexibility enhances its applicability to diverse experimental settings, including cases in which high-quality or finely annotated reference data are not available [24]. Furthermore, an important line of evidence supporting the accuracy of Cell2Map comes from the spatial expression patterns of lineage-specific marker genes across multiple cell types and independent studies. For example, tumor-associated markers (e.g. ERBB2 [47], HOXB2 [46], FOXA1 [60]) were enriched within histologically defined malignant regions, whereas markers (e.g. PTPRC [49], CD3D [50], ANKRD30A [61]) for normal epithelial or immune populations (e.g. NK/T cells) were predominantly localized outside tumor cores or within stromal and peritumoral regions. The strong agreement between projected cell locations and independently observed marker-gene expression provides orthogonal biological validation that the reconstructed maps are not merely computationally plausible, but are also consistent with tissue morphology and known cell-type-specific transcriptional programs. These validations establish Cell2Map as a sensitive, accurate, and robust framework for integrating scRNA-seq and SRT data, enabling improved spatial resolution of cellular organization and providing a valuable tool for studying complex tissues and disease microenvironments.

One limitation of the current study is that the benchmark simulations mainly evaluate Cell2Map under settings in which the scRNA-seq reference contains the major cell populations present in the spatial transcriptomics data. In real applications, however, the scRNA-seq reference may be incomplete, and some cell types may be missing or under-represented in the reference dataset. Therefore, if a cell type is completely absent from the reference, Cell2Map may not be able to explicitly identify that missing population and may instead assign those cells to transcriptionally similar cell types. Similarly, when rare cell populations are strongly under-represented in the reference, the accuracy of their spatial mapping may decrease. Future work will include systematic benchmarking under incomplete-reference scenarios, including removing selected cell types from the reference, down-sampling rare populations, and evaluating robustness across different donors, batches, and tissue conditions.

Several directions may further improve Cell2Map and extend its applicability. First, the current framework could be enhanced by incorporating additional biological priors, such as known cell-cell interaction networks [62], ligand receptor signaling [63], or histology-derived features (e.g. morphology or texture from H&E images) [64], to provide complementary constraints beyond transcriptional and spatial proximity. Second, methodologically, future versions of Cell2Map could explore more expressive latent representations, including hierarchical or multi-resolution embeddings that explicitly model spatial structure at different scales. Integrating uncertainty estimation may further reduce false positives by quantifying confidence in cell-to-spot assignments. In the current framework, the soft mapping matrix provides a natural basis for per-cell uncertainty quantification: the entropy of each cell’s assignment distribution can be used to flag ambiguous assignments, especially under high noise or low spot resolution. Cells with high entropy may benefit from follow-up analysis or could be flagged as uncertain in downstream workflows. Third, Cell2Map uses fixed weighting coefficients to maintain interpretability, reproducibility, and ease of implementation. Future work may incorporate adaptive or data-driven weighting strategies, such as Bayesian optimization, uncertainty-based weighting, multi-objective learning, or attention-based parameter estimation, to automatically balance different objective terms according to dataset-specific characteristics. In addition, extending the framework to jointly integrate multiple spatial modalities (e.g. single-cell spatial proteomics [65, 66] or imaging-based transcriptomics [67]) and to leverage transfer learning or foundation models trained on large spatial datasets could substantially improve performance in data-limited settings. Implementation of these directions will further advance Cell2Map into a more general and extensible platform for spatial multi-omics integration.

**Key Points**

- Cell2Map is an unsupervised deep learning method that accurately maps single-cell RNA-seq profiles to spatial transcriptomics spots for high-resolution cell localization.
- Cell2Map uses the graph attention autoencoder architecture with a four-term objective function to jointly optimize transcriptional similarity, spatial structure, cell density, and embedding-level relationships.
- Cell2Map accurately reconstructs tumor subclones, fibrosis-associated cell localization, and tumor microenvironments, aligning closely with histological annotations and marker-gene expression patterns.

## Supplementary Material

Supplementary_File1_6_9_bbag404

## Data Availability

The simulated data of mouse cerebellum and hippocampus can be obtained from https://github.com/digitalcytometry/cytospace. The scRNA-seq data of DCIS1 and DCIS2 of ductal carcinoma can be obtained from the National Center for Biotechnology Information Gene Expression Omnibus (NCBI GEO) with accession numbers GSM5493629 and GSM5493631, respectively. The SRT data of DCIS1 and DCIS2 can be obtained from the Spatial Transcript Omics DataBase with accession numbers STSP0000956 and STSP0000958, respectively. The HER2+ breast cancer scRNA-seq data can be obtained from the GEO with accession number GSE176078. The Visium breast cancer SRT data are available in https://www.10xgenomics.com/datasets/human-breast-cancer-visium-fresh-frozen-whole-transcriptome-1-standard. The MI scRNA-seq data can be obtained from the GEO with accession number GSE129175. The SRT data of MI can be obtained from the GEO with accession number GSE165857. The code for Cell2Map is available at https://github.com/shaoqiangzhang/Cell2Map.
