# Supplementary material for: Navigating cell maps by deep learning integration of single-cell and spatially resolved transcriptomics
Source: Brief Bioinform. 2026 Jul 30;27(4):bbag404. doi: 10.1093/bib/bbag404 (PMC13435232; doi:10.1093/bib/bbag404)
Supplement: Supplementary file 1 [file supplementary_file1_6_9_bbag404.docx]

# SUPPLEMENTARY DATA

# Navigating Cell Maps by Deep Learning Integration of Single-Cell and Spatially Resolved Transcriptomics

Yanan Chen^1^, Ruoyu Chen^2^, Shaoqiang Zhang^1*^, Yong Chen^3*^

^1^ College of Computer and Information Engineering, Tianjin Normal University, Tianjin, Tianjin 300387, China

^2^ College of Arts and Sciences, University of Pennsylvania, Philadelphia, PA 19104, USA

^3^ Department of Biological and Biomedical Sciences, Rowan University, Glassboro, NJ 08028, USA

*To whom correspondence should be addressed: Shaoqiang Zhang, Tel: +86 186 4900 6931, Email: [zhangshaoqiang@tjnu.edu.cn](mailto:zhangshaoqiang@tjnu.edu.cn); Yong Chen, Tel: +1 856 256 4500 ext. 53589; Email: [chenyong@rowan.edu](mailto:chenyong@rowan.edu)

**Supplementary File1** includes Table S1-S20 and Figure S1-S6.

**Table S1**. Summary of simulated benchmark datasets used in this study.

| **Datasets** | **Species** | **Cell types (#cells)** | **Resolution (cells/spot)** | **Noise level** | **#Spots** | **#Genes** |
| --- | --- | --- | --- | --- | --- | --- |
| Cerebellum | Mouse | Granule (11931),  Bergmann (2539),  Purkinje (2184),  Oligodendrocyte (1417),  Pvalb (1226),  Fibroblast (996),  Astrocyte (755),  Endothelial (683),  Microglia (589),  Choroid (545),  Nnat (507) | 5 | 5%  10%  25% | 1709 | 15928 |
|  |  |  | 15 | 5%  10%  25% | 570 | 15928 |
|  |  |  | 30 | 5%  10%  25% | 285 | 15928 |
| Hippocampus | Mouse | Cajal Retzius (6989),  Neurogenesis (2848),  Dentate (2820),  Astrocyte (1626),  Ependyma (1617),  Sic17ab (1444),  Microglia (1407),  Polydendrocyte (1141),  Entorhinal (781),  Endothelial (608),  Choroid (552),  Gad2 (461),  Fibroblast (448),  CA1 (270),  Oligodendrocyte (193),  CA2CA3 (87), Mural (80) | 5 | 5%  10%  25% | 1453 | 14562 |
|  |  |  | 15 | 5%  10%  25% | 484 | 14562 |
|  |  |  | 30 | 5%  10%  25% | 242 | 14562 |

**Table S2**. Summary of real datasets used in this study.

| **Dataset** | **Species** | **Issue** | **Pathological state** | **#Cells** | **#Spots** | **#Shared genes** | **#Cell types** |
| --- | --- | --- | --- | --- | --- | --- | --- |
| MI | Mouse | Heart | myocardial infarction | 11492 | 2835 | 12364 | 5 |
| DCIS1 | Human | Breast | Human ductal carcinoma *in situ* | 6828 | 1567 | 8742 | 12 |
| DCIS2 | Human | Breast | DCIS+ invasive | 3748 | 2063 | 8742 | 10 |
| Breast  Cancer | Human | Breast | HER2^+^ breast cancer | 24271 | 3798 | 16854 | 18 |

**Table S3.** ACC sensitivity of Cell2Map for different $\lambda₁$and λ₂ settings with fixed $\lambda_{g}= 1, \lambda_{d}= 8$ and $\alpha=7$on simulated benchmark data with 5% noise.

| **Setting** | | **Mouse Cerebellum** | | | **Mouse Hippocampus** | | |
| --- | --- | --- | --- | --- | --- | --- | --- |
| $\lambda₁$ | $\lambda₂$ | 5-cell-per-spot | 15-cell-per-spot | 30-cell-per-spot | 5-cell-per-spot | 15-cell-per-spot | 30-cell-per-spot |
| 0.001 | 0.3 | 0.8047 | 0.7154 | 0.6287 | 0.6282 | 0.3814 | 0.1977 |
|  | 0.5 | 0.9271 | 0.3932 | 0.2796 | 0.6724 | 0.3729 | 0.2288 |
|  | 0.7 | 0.7232 | 0.6304 | 0.2842 | 0.6540 | 0.3585 | 0.0926 |
|  | 0.9 | 0.7182 | 0.5466 | 0.2854 | 0.6646 | 0.3711 | 0.2264 |
| 0.01 | 0.3 | 0.8081 | 0.5318 | 0.4208 | 0.6232 | 0.3741 | 0.2437 |
|  | 0.5 | 0.8041 | 0.6318 | 0.5318 | 0.6619 | 0.3615 | 0.2078 |
|  | 0.7 | **0.9356** | **0.8091** | **0.7595** | **0.6848** | **0.4277** | **0.3098** |
|  | 0.9 | 0.9201 | 0.5994 | 0.2542 | 0.6601 | 0.3244 | 0.1459 |
| 0.1 | 0.3 | 0.6931 | 0.4388 | 0.2580 | 0.6764 | 0.1278 | 0.1083 |
|  | 0.5 | 0.6311 | 0.4482 | 0.2796 | 0.6141 | 0.2115 | 0.0874 |
|  | 0.7 | 0.9312 | 0.4845 | 0.2845 | 0.6790 | 0.1416 | 0.0754 |
|  | 0.9 | 0.6988 | 0.5996 | 0.3622 | 0.4848 | 0.1753 | 0.0499 |
| 1 | 0.3 | 0.1208 | 0.0305 | 0.0121 | 0.0212 | 0.0054 | 0.0031 |
|  | 0.5 | 0.1511 | 0.0642 | 0.0176 | 0.0597 | 0.0089 | 0.0038 |
|  | 0.7 | 0.2426 | 0.0920 | 0.0540 | 0.0665 | 0.0106 | 0.0040 |
|  | 0.9 | 0.4994 | 0.1741 | 0.1151 | 0.0329 | 0.0090 | 0.0041 |

**Table S4.** ACC sensitivity of Cell2Map for different $\lambda_{d}$ and $\alpha$ settings with fixed $\lambda_{1}= 0.01$ and $\lambda_{2}=0.7$on simulated benchmark data with 5% noise.

| **Setting** | | **Mouse Cerebellum** | | | **Mouse Hippocampus** | | |
| --- | --- | --- | --- | --- | --- | --- | --- |
| $\lambda_{d}$ | $\alpha$ | 5-cell-per-spot | 15-cell-per-spot | 30-cell-per-spot | 5-cell-per-spot | 15-cell-per-spot | 30-cell-per-spot |
| 1 | 1 | 0.9236 | 0.3798 | 0.1642 | 0.4762 | 0.3683 | 0.2013 |
|  | 3 | 0.5725 | 0.4019 | 0.2855 | 0.6847 | 0.3372 | 0.3044 |
|  | 5 | 0.9347 | 0.6230 | 0.3554 | 0.5905 | 0.3364 | 0.3077 |
|  | 7 | 0.8924 | 0.6432 | 0.2535 | 0.6810 | 0.3679 | 0.1629 |
|  | 9 | 0.9340 | 0.8079 | 0.6487 | 0.6293 | 0.3642 | 0.2067 |
| 2 | 1 | 0.9226 | 0.7001 | 0.3877 | 0.4945 | 0.1268 | 0.0533 |
|  | 3 | 0.9276 | 0.8085 | 0.4631 | 0.6412 | 0.3822 | 0.1943 |
|  | 5 | 0.9325 | 0.8086 | 0.5928 | 0.6846 | 0.4064 | 0.1426 |
|  | 7 | 0.9082 | 0.8048 | 0.6480 | 0.6803 | 0.3763 | 0.0963 |
|  | 9 | 0.9340 | 0.8061 | 0.6550 | 0.6703 | 0.3852 | 0.2107 |
| 4 | 1 | 0.6014 | 0.3852 | 0.2913 | 0.4978 | 0.0147 | 0.0048 |
|  | 3 | 0.9286 | 0.8061 | 0.6118 | 0.6207 | 0.2405 | 0.1345 |
|  | 5 | 0.9313 | 0.8090 | 0.6475 | 0.6049 | 0.3835 | 0.2197 |
|  | 7 | 0.9248 | 0.8044 | 0.6546 | 0.6822 | 0.3287 | 0.0739 |
|  | 9 | 0.9317 | 0.80 | 0.6821 | 0.6021 | 0.4043 | 0.2230 |
| 8 | 1 | 0.8862 | 0.3626 | 0.1792 | 0.4110 | 0.0139 | 0.0051 |
|  | 3 | 0.9094 | 0.8048 | 0.5555 | 0.5561 | 0.1173 | 0.0498 |
|  | 5 | 0.9276 | 0.8050 | 0.6607 | 0.6701 | 0.2390 | 0.0405 |
|  | 7 | **0.9356** | **0.8091** | **0.7595** | **0.6848** | **0.4277** | **0.3098** |
|  | 9 | 0.9263 | 0.8009 | 0.6655 | 0.6021 | 0.3125 | 0.2260 |
| 16 | 1 | 0.6049 | 0.4783 | 0.0534 | 0.4682 | 0.0128 | 0.0033 |
|  | 3 | 0.6838 | 0.4542 | 0.3549 | 0.6436 | 0.3646 | 0.0192 |
|  | 5 | 0.8005 | 0.6840 | 0.6452 | 0.6782 | 0.2180 | 0.0634 |
|  | 7 | 0.9267 | 0.8001 | 0.6592 | 0.6480 | 0.2516 | 0.1219 |
|  | 9 | 0.9278 | 0.8024 | 0.6657 | 0.5745 | 0.2177 | 0.1046 |

**Table S5.** $\lambda$₁ and $\lambda₂$ values used for each real dataset.

| Dataset | $\boldsymbol{\lambda}\mathbf{₁}$ | $\boldsymbol{\lambda}\mathbf{₂}$ |
| --- | --- | --- |
| DCIS1 (mapping to DCIS1 SRT) | 0.01 | 0.7 |
| DCIS2 | 0.01 | 0.65 |
| DCIS1 scRNA-seq mapping to breast cancer SRT | 0.02 | 0.4 |
| HER2+ scRNA-seq mapping to breast cancer SRT | 0.01 | 0.7 |

**Table S6.** Sensitivity of Cell2Map to the number of KNN neighbors $k$.

| $\boldsymbol{k}$ | **3** | **5** | **6(default)** | **8** | **10** |
| --- | --- | --- | --- | --- | --- |
| $ACC$ | 0.9123 | 0.9298 | **0.9356** | 0.9341 | 0.9276 |
| $Mean {Pr}_{c2s}$ | 0.8964 | 0.9171 | **0.9215** | 0.9202 | 0.9135 |

**Table S7**. Sensitivity of Cell2Map to the spatial neighbor radius.

| **Radius** | **3** | **5(default)** | **7** | **10** |
| --- | --- | --- | --- | --- |
| $ACC$ | 0.9214 | **0.9356** | 0.9332 | 0.9281 |
| $Mean {Pr}_{c2s}$ | 0.9072 | **0.9215** | 0.9197 | 0.9143 |

**Table S8**. Sensitivity of Cell2Map to the learning rate.

| **Learning rate** | **0.01** | **0.05** | **0.1(default)** | **0.2** |
| --- | --- | --- | --- | --- |
| $ACC$ | 0.9187 | 0.9305 | **0.9356** | 0.9223 |
| $Mean {Pr}_{c2s}$ | 0.9046 | 0.9173 | **0.9215** | 0.9091 |

**Table S9.** The mean ACC scores (with standard deviations) of four cell-to-spot alignment algorithms on the simulated mouse cerebellum dataset.

| **Cell density** | **5 cells per spot** | | | **15 cells per spot** | | | **30 cells per spot** | | |
| --- | --- | --- | --- | --- | --- | --- | --- | --- | --- |
| Noise level | 5% | 10% | 25% | 5% | 10% | 25% | 5% | 10% | 25% |
| Cell2Map | **0.9356**  (0.002) | **0.9239**  (0.002) | **0.5282**  (0.014) | **0.8091**  (0.017) | **0.7595**  (0.008) | **0.2828**  (0.009) | **0.4997**  (0.003) | **0.4301**  (0.004) | **0.2483**  (0.006) |
| Celloc | 0.6223  (0.003) | 0.5562  (0.002) | 0.3488  (0.002) | 0.2601  (0.001) | 0.2198  (0.001) | 0.1888  (0.014) | 0.2554  (0.001) | 0.1739  (0.002) | 0.1141  (0.004) |
| Cytospace | 0.6189  (0.003) | 0.5324  (0.002) | 0.4103  (0.003) | 0.5824  (0.004) | 0.4514  (0.003) | 0.2713  (0.004) | 0.4536  (0.002) | 0.3504  (0.002) | 0.2142  (0.002) |
| Tangram | 0.6201  (0.002) | 0.5239  (0.002) | 0.3709  (0.003) | 0.5226  (0.002) | 0.4191  (0.003) | 0.2707  (0.002) | 0.4055  (0.002) | 0.3103  (0.002) | 0.1879  (0.002) |

**Table S10.** The mean ACC scores (with standard deviations) of four cell-to-spot alignment algorithms on the simulated mouse hippocampus dataset.

| **Cell density** | **5 cells per spot** | | | **15 cells per spot** | | | **30 cells per spot** | | |
| --- | --- | --- | --- | --- | --- | --- | --- | --- | --- |
| Noise level | 5% | 10% | 25% | 5% | 10% | 25% | 5% | 10% | 25% |
| Cell2Map | **0.6848**  (0.008) | **0.5471**  (0.019) | **0.4231**  (0.005) | **0.4277**  (0.004) | **0.3818**  (0.012) | **0.2344**  (0.007) | **0.3098**  (0.002) | **0.2320**  (0.008) | **0.1442**  (0.007) |
| Celloc | 0.5388  (0.002) | 0.4795  (0.002) | 0.3210  (0.007) | 0.3037  (0.001) | 0.2410  (0.001) | 0.1563  (0.002) | 0.1997  (0.001) | 0.1429  (0.001) | 0.0892  (0.004) |
| Cytospace | 0.5203  (0.003) | 0.4536  (0.003) | 0.3256  (0.004) | 0.4003  (0.003) | 0.3405  (0.004) | 0.2232  (0.004) | 0.2903  (0.003) | 0.2231  (0.003) | 0.1404  (0.002) |
| Tangram | 0.5171  (0.004) | 0.4410  (0.002) | 0.3214  (0.003) | 0.3891  (0.003) | 0.3167  (0.002) | 0.2158  (0.002) | 0.2806  (0.002) | 0.2198  (0.001) | 0.1425  (0.001) |

**Table S11.** The mean $Pr_{c2s}$ scores (with standard deviations) of 11 cell types on the simulated mouse cerebellum dataset.

| **Cell density** | | **5 cells per spot** | | | **15 cells per spot** | | | **30 cells per spot** | | |
| --- | --- | --- | --- | --- | --- | --- | --- | --- | --- | --- |
| Cell type | Noise level | 5% | 10% | 25% | 5% | 10% | 25% | 5% | 10% | 25% |
| Astrocyte | Cell2Map | **0.9296**  (0.003) | **0.9139**  (0.003) | **0.5873**  (0.009) | **0.8219**  (0.021) | **0.7918**  (0.013) | **0.4543**  (0.036) | 0.4975  (0.007) | **0.5117**  (0.006) | **0.4095**  (0.015) |
|  | Celloc | 0.6569  (0.004) | 0.5991  (0.002) | 0.3778  (0.205) | 0.5488  (0.004) | 0.4573  (0.005) | 0.4139  (0.015) | **0.5633**  (0.003) | 0.4973  (0.007) | 0.4227  (0.025) |
|  | Cytospace | 0.6412  (0.003) | 0.5128  (0.003) | 0.4136  (0.004) | 0.5604  (0.004) | 0.5030  (0.006) | 0.3624  (0.007) | 0.5517  (0.003) | 0.4527  (0.006) | 0.2717  (0.011) |
|  | Tangram | 0.5928  (0.013) | 0.5076  (0.014) | 0.3969  (0.011) | 0.5641  (0.011) | 0.4798  (0.007) | 0.3423  (0.022) | 0.5130  (0.012) | 0.4175  (0.010) | 0.2579  (0.015) |
| Bergmann | Cell2Map | **0.9286**  (0.001) | **0.9298**  (0.001) | **0.6377**  (0.015) | **0.7079**  (0.035) | **0.6601**  (0.018) | **0.5951**  (0.050) | 0.2992  (0.006) | 0.3124  (0.008) | **0.3875**  (0.008) |
|  | Celloc | 0.7135  (0.005) | 0.6642  (0.003) | 0.5196  (0.008) | 0.5464  (0.004) | 0.5003  (0.005) | 0.4042  (0.047) | **0.5205**  (0.007) | 0.3421  (0.114) | 0.1598  (0.134) |
|  | Cytospace | 0.7014  (0.003) | 0.5511  (0.003) | 0.4204  (0.007) | 0.5526  (0.004) | 0.4532  (0.005) | 0.3375  (0.006) | 0.4622  (0.005) | **0.3616**  (0.004) | 0.2433  (0.006) |
|  | Tangram | 0.6194  (0.007) | 0.5365  (0.013) | 0.4153  (0.008) | 0.5497  (0.006) | 0.4696  (0.004) | 0.3223  (0.008) | 0.4630  (0.007) | 0.3597  (0.004) | 0.2347  (0.003) |
| Choroid | Cell2Map | **0.8634**  (0.007) | **0.8688**  (0.003) | 0.2729  (0.007) | **0.6899**  (0.020) | **0.7131**  (0.014) | 0.1207  (0.004) | 0.3834  (0.011) | 0.3576  (0.007) | 0.2623  (0.012) |
|  | Celloc | 0.3060  (0.002) | 0.2822  (0.003) | 0.2458  (0.005) | 0.1632  (0.003) | 0.1418  (0.002) | 0.1237  (0.007) | 0.1296  (0.003) | 0.1224  (0.002) | 0.0903  (0.003) |
|  | Cytospace | 0.2942  (0.005) | 0.2834  (0.006) | 0.2492  (0.006) | 0.6754  (0.003) | 0.5517  (0.005) | **0.3782**  (0.005) | **0.6021**  (0.004) | 0.4563  (0.004) | **0.3128**  (0.009) |
|  | Tangram | 0.7147  (0.010) | 0.5708  (0.016) | **0.3822**  (0.021) | 0.6818  (0.011) | 0.5383  (0.010) | 0.3489  (0.015) | 0.5963  (0.010) | **0.4682**  (0.013) | 0.2748  (0.014) |
| Endothelial | Cell2Map | **0.9221**  (0.001) | **0.9241**  (0.002) | **0.4891**  (0.007) | **0.8784**  (0.014) | **0.8727**  (0.012) | 0.3677  (0.012) | 0.5503  (0.008) | **0.6034**  (0.010) | **0.4566**  (0.010) |
|  | Celloc | 0.5624  (0.003) | 0.5152  (0.004) | 0.4611  (0.009) | 0.3824  (0.004) | 0.3549  (0.004) | 0.3227  (0.015) | 0.3528  (0.004) | 0.3127  (0.002) | 0.2367  (0.010) |
|  | Cytospace | 0.5437  (0.003) | 0.5173  (0.004) | 0.4173  (0.009) | 0.6032  (0.004) | 0.5123  (0.004) | **0.4019**  (0.004) | 0.5447  (0.005) | 0.5244  (0.005) | 0.3927  (0.007) |
|  | Tangram | 0.5877  (0.015) | 0.5075  (0.014) | 0.3917  (0.018) | 0.5801  (0.009) | 0.5128  (0.015) | 0.3935  (0.017) | **0.5988**  (0.013) | 0.5089  (0.012) | 0.3716  (0.015) |
| Fibroblast | Cell2Map | **0.9078**  (0.003) | **0.9056**  (0.002) | **0.3929**  (0.010) | **0.8543**  (0.011) | **0.8370**  (0.008) | 0.2218  (0.004) | 0.4859  (0.003) | **0.5004**  (0.008) | **0.3473**  (0.008) |
|  | Celloc | 0.4698  (0.005) | 0.4194  (0.003) | 0.3040  (0.091) | 0.2723  (0.003) | 0.2429  (0.002) | 0.2036  (0.010) | 0.2324  (0.002) | 0.2050  (0.003) | 0.1780  (0.004) |
|  | Cytospace | 0.5077  (0.004) | 0.4938  (0.004) | 0.3722  (0.008) | 0.6203  (0.005) | 0.4976  (0.004) | **0.4123**  (0.006) | **0.6102**  (0.010) | 0.4836  (0.005) | 0.3140  (0.006) |
|  | Tangram | 0.6428  (0.010) | 0.5083  (0.012) | 0.3658  (0.009) | 0.6136  (0.007) | 0.5091  (0.011) | 0.3658  (0.017) | 0.5833  (0.006) | 0.4798  (0.012) | 0.3225  (0.010) |
| Granule | Cell2Map | **0.9525**  (0.002) | **0.9339**  (0.002) | **0.6743**  (0.020) | **0.8601**  (0.013) | **0.7953**  (0.008) | **0.4025**  (0.035) | **0.5828**  (0.003) | **0.4604**  (0.004) | 0.1721  (0.007) |
|  | Celloc | 0.7968  (0.004) | 0.7271  (0.004) | 0.5764  (0.012) | 0.5208  (0.005) | 0.4257  (0.004) | 0.3181  (0.014) | 0.4263  (0.004) | 0.3260  (0.005) | **0.2066**  (0.006) |
|  | Cytospace | 0.7530  (0.003) | 0.5304  (0.003) | 0.4325  (0.009) | 0.5517  (0.006) | 0.3836  (0.005) | 0.2533  (0.006) | 0.4327  (0.006) | 0.2342  (0.002) | 0.1625  (0.003) |
|  | Tangram | 0.6226  (0.002) | 0.5367  (0.002) | 0.3827  (0.006) | 0.4815  (0.004) | 0.3766  (0.004) | 0.2293  (0.004) | 0.3204  (0.003) | 0.2284  (0.002) | 0.1279  (0.003) |
| Microglia | Cell2Map | **0.9485**  (0.001) | **0.9447**  (0.003) | **0.5366**  (0.019) | **0.8359**  (0.019) | **0.7911**  (0.005) | 0.2784  (0.020) | 0.4818  (0.008) | **0.4800**  (0.011) | **0.2865**  (0.008) |
|  | Celloc | 0.6299  (0.006) | 0.5472  (0.010) | 0.3340  (0.146) | 0.3482  (0.003) | 0.3250  (0.005) | 0.2749  (0.020) | 0.2470  (0.003) | 0.2387  (0.004) | 0.2073  (0.009) |
|  | Cytospace | 0.6318  (0.002) | 0.4549  (0.003) | 0.3981  (0.007) | 0.5571  (0.004) | 0.4509  (0.005) | **0.3133**  (0.007) | 0.4526  (0.004) | 0.3921  (0.003) | 0.2805  (0.007) |
|  | Tangram | 0.6237  (0.019) | 0.4863  (0.016) | 0.3115  (0.015) | 0.5592  (0.016) | 0.4521  (0.016) | 0.3099  (0.016) | **0.4960**  (0.015) | 0.4110  (0.007) | 0.2488  (0.009) |
| Nnat | Cell2Map | **0.9092**  (0.003) | **0.8965**  (0.002) | **0.3392**  (0.008) | **0.8382**  (0.021) | **0.7892**  (0.020) | 0.3051  (0.021) | **0.5228**  (0.009) | **0.4482**  (0.006) | 0.2865  (0.012) |
|  | Celloc | 0.4320  (0.006) | 0.3694  (0.003) | 0.3128  (0.011) | 0.3730  (0.006) | 0.2947  (0.005) | 0.2362  (0.008) | 0.3461  (0.004) | 0.2656  (0.007) | 0.2149  (0.004) |
|  | Cytospace | 0.4271  (0.005) | 0.4083  (0.005) | 0.3901  (0.010) | 0.6277  (0.006) | 0.4921  (0.005) | 0.3305  (0.011) | 0.4536  (0.006) | 0.4282  (0.004) | **0.3164**  (0.011) |
|  | Tangram | 0.6250  (0.018) | 0.5137  (0.025) | 0.3467  (0.013) | 0.6056  (0.012) | 0.4778  (0.013) | **0.3425**  (0.009) | 0.5216  (0.011) | 0.4297  (0.016) | 0.2881  (0.009) |
| Oligo-  dendrocyte | Cell2Map | **0.9177**  (0.003) | **0.9182**  (0.002) | **0.4639**  (0.010) | **0.7909**  (0.013) | **0.7700**  (0.007) | **0.3700**  (0.016) | 0.4877  (0.007) | **0.4472**  (0.004) | **0.3033**  (0.008) |
|  | Celloc | 0.5415  (0.005) | 0.4720  (0.004) | 0.4133  (0.006) | 0.3831  (0.003) | 0.3284  (0.005) | 0.2656  (0.013) | 0.3282  (0.003) | 0.2731  (0.004) | 0.1844  (0.017) |
|  | Cytospace | 0.5325  (0.003) | 0.5314  (0.004) | 0.3874  (0.007) | 0.5843  (0.003) | 0.4822  (0.005) | 0.3367  (0.006) | **0.5043**  (0.004) | 0.3810  (0.003) | 0.2979  (0.007) |
|  | Tangram | 0.6157  (0.011) | 0.5009  (0.014) | 0.3557  (0.014) | 0.5717  (0.008) | 0.4609  (0.011) | 0.3158  (0.009) | 0.4941  (0.007) | 0.3970  (0.008) | 0.2691  (0.008) |
| Purkinje | Cell2Map | **0.9075**  (0.001) | **0.8923**  (0.003) | **0.4718**  (0.028) | **0.5964**  (0.027) | **0.5455**  (0.010) | 0.1414  (0.010) | **0.2621**  (0.014) | **0.2521**  (0.003) | 0.1323  (0.005) |
|  | Celloc | 0.5586  (0.009) | 0.4893  (0.005) | 0.1542  (0.052) | 0.0578  (0.001) | 0.0483  (0.001) | 0.0507  (0.011) | 0.1006  (0.002) | 0.0387  (0.001) | 0.0298  (0.005) |
|  | Cytospace | 0.5514  (0.002) | 0.4587  (0.003) | 0.3133  (0.014) | 0.5483  (0.003) | 0.3296  (0.003) | **0.1874**  (0.005) | 0.2541  (0.003) | 0.1521  (0.001) | **0.1529**  (0.005) |
|  | Tangram | 0.5662  (0.014) | 0.4489  (0.010) | 0.2597  (0.010) | 0.4128  (0.009) | 0.2971  (0.011) | 0.1677  (0.012) | 0.2610  (0.005) | 0.1942  (0.007) | 0.1106  (0.005) |
| Pvalb | Cell2Map | **0.9296**  (0.003) | **0.9212**  (0.003) | **0.4597**  (0.013) | **0.8191**  (0.017) | **0.7775**  (0.010) | 0.3221  (0.018) | 0.5288  (0.011) | **0.4927**  (0.005) | **0.3126**  (0.025) |
|  | Celloc | 0.5576  (0.008) | 0.4863  (0.005) | 0.3972  (0.017) | 0.3678  (0.006) | 0.3050  (0.001) | 0.2241  (0.010) | 0.3471  (0.007) | 0.2701  (0.005) | 0.1747  (0.012) |
|  | Cytospace | 0.5614  (0.004) | 0.5433  (0.004) | 0.4523  (0.009) | 0.6032  (0.005) | 0.5341  (0.003) | **0.3422**  (0.004) | **0.5532**  (0.005) | 0.4324  (0.007) | 0.2824  (0.006) |
|  | Tangram | 0.6474  (0.013) | 0.5619  (0.012) | 0.3874  (0.014) | 0.5891  (0.006) | 0.4860  (0.009) | 0.3280  (0.011) | 0.5119  (0.007) | 0.4147  (0.011) | 0.2617  (0.009) |

**Table S12.** The mean $Pr_{c2s}$ scores (with standard deviations) of 17 cell types on the simulated mouse hippocampus dataset.

| **Cell density** | | **5 cells per spot** | | | **15 cells per spot** | | | **30 cells per spot** | | |
| --- | --- | --- | --- | --- | --- | --- | --- | --- | --- | --- |
| Cell type | Noise level | 5% | 10% | 25% | 5% | 10% | 25% | 5% | 10% | 25% |
| Astrocyte | Cell2Map | **0.9309**  (0.015) | **0.8721**  (0.010) | **0.7860**  (0.011) | 0.7323  (0.010) | **0.8093**  (0.022) | 0.5289  (0.043) | 0.3601  (0.004) | 0.2452  (0.004) | 0.1291  (0.012) |
|  | Celloc | 0.9071  (0.003) | 0.8472  (0.006) | 0.3364  (0.274) | **0.7960**  (0.005) | 0.7347  (0.007) | **0.5633**  (0.035) | **0.6437**  (0.028) | **0.5286**  (0.014) | **0.3790**  (0.020) |
|  | Cytospace | 0.5320  (0.004) | 0.4134  (0.003) | 0.2727  (0.003) | 0.2904  (0.004) | 0.2231  (0.004) | 0.1324  (0.005) | 0.1743  (0.004) | 0.1264  (0.004) | 0.0813  (0.005) |
|  | Tangram | 0.4579  (0.010) | 0.4021  (0.016) | 0.2855  (0.010) | 0.2699  (0.008) | 0.2182  (0.009) | 0.1387  (0.006) | 0.1619  (0.005) | 0.1157  (0.004) | 0.0735  (0.004) |
| CA1 | Cell2Map | 0.6285  (0.031) | 0.5549  (0.030) | 0.3995  (0.020) | **0.7537**  (0.026) | 0.5216  (0.044) | 0.3106  (0.067) | 0.3837  (0.004) | 0.2848  (0.020) | 0.1843  (0.024) |
|  | Celloc | 0.4374  (0.002) | 0.3984  (0.002) | 0.3411  (0.007) | 0.2974  (0.004) | 0.2550  (0.004) | 0.1666  (0.009) | 0.2414  (0.006) | 0.1958  (0.004) | 0.1087  (0.009) |
|  | Cytospace | **0.7911**  (0.010) | **0.7232**  (0.009) | 0.6013  (0.008) | 0.6922  (0.009) | **0.6527**  (0.010) | **0.5613**  (0.011) | 0.6364  (0.014) | **0.5911**  (0.013) | **0.4752**  (0.019) |
|  | Tangram | 0.7079  (0.012) | 0.6637  (0.023) | **0.6171**  (0.023) | 0.6986  (0.018) | 0.6352  (0.018) | 0.5268  (0.030) | **0.6515**  (0.023) | 0.5878  (0.020) | 0.4679  (0.023) |
| CA2CA3 | Cell2Map | 0.7340  (0.016) | **0.7436**  (0.068) | **0.6895**  (0.035) | 0.4733  (0.028) | 0.3746  (0.040) | **0.5543**  (0.088) | 0.3411  (0.015) | 0.4029  (0.027) | **0.3748**  (0.039) |
|  | Celloc | 0.7596  (0.008) | 0.6613  (0.002) | 0.4292  (0.033) | **0.6724**  (0.010) | **0.5186**  (0.020) | 0.1332  (0.018) | 0.4356  (0.011) | 0.1958  (0.013) | 0.0774  (0.011) |
|  | Cytospace | **0.7624**  (0.011) | 0.6112  (0.006) | 0.5350  (0.005) | 0.6034  (0.010) | 0.5130  (0.011) | 0.5153  (0.012) | **0.5730**  (0.021) | **0.5932**  (0.023) | 0.3512  (0.027) |
|  | Tangram | 0.7422  (0.036) | 0.5896  (0.036) | 0.6171  (0.052) | 0.5705  (0.033) | 0.4791  (0.031) | 0.4630  (0.045) | 0.4812  (0.046) | 0.5878  (0.025) | 0.3691  (0.035) |
| Cajal  Retzius | Cell2Map | **0.6785**  (0.025) | **0.4629**  (0.056) | **0.3412**  (0.016) | **0.2983**  (0.009) | **0.2116**  (0.017) | **0.1276**  (0.015) | **0.1814**  (0.001) | **0.1200**  (0.006) | **0.0604**  (0.005) |
|  | Celloc | 0.5341  (0.008) | 0.4522  (0.005) | 0.3343  (0.008) | 0.2926  (0.002) | 0.1952  (0.002) | 0.0896  (0.015) | 0.1220  (0.002) | 0.0568  (0.002) | 0.0216  (0.003) |
|  | Cytospace | 0.4519  (0.006) | 0.3320  (0.003) | 0.2138  (0.003) | 0.2113  (0.003) | 0.1863  (0.004) | 0.1175  (0.003) | 0.1132  (0.002) | 0.0927  (0.002) | 0.0574  (0.002) |
|  | Tangram | 0.4064  (0.006) | 0.3238  (0.005) | 0.2100  (0.005) | 0.2044  (0.003) | 0.1613  (0.003) | 0.0979  (0.003) | 0.1020  (0.002) | 0.0805  (0.002) | 0.0533  (0.003) |
| Choroid | Cell2Map | 0.5475  (0.028) | 0.4680  (0.042) | 0.3730  (0.008) | 0.1368  (0.007) | 0.2137  (0.014) | 0.2368  (0.031) | 0.4123  (0.006) | 0.2701  (0.014) | 0.2347  (0.013) |
|  | Celloc | 0.4200  (0.004) | 0.3871  (0.003) | 0.3394  (0.005) | 0.2432  (0.001) | 0.2227  (0.002) | 0.1637  (0.015) | 0.1811  (0.004) | 0.1532  (0.003) | 0.1163  (0.007) |
|  | Cytospace | **0.8325**  (0.004) | **0.8275**  (0.004) | 0**.7136**  (0.003) | **0.8219**  (0.018) | **0.8174**  (0.019) | **0.7216**  (0.017) | **0.7548**  (0.005) | **0.7137**  (0.007) | **0.6143**  (0.006) |
|  | Tangram | 0.8288  (0.019) | 0.8040  (0.013) | 0.6755  (0.028) | 0.8175  (0.020) | 0.8056  (0.010) | 0.6973  (0.015) | 0.7435  (0.012) | 0.7012  (0.014) | 0.6019  (0.020) |
| Dentate | Cell2Map | **0.6524**  (0.009) | **0.5351**  (0.024) | **0.3835**  (0.005) | **0.5855**  (0.014) | **0.4646**  (0.020) | **0.3785**  (0.013) | **0.3537**  (0.004) | **0.3801**  (0.010) | **0.3348**  (0.013) |
|  | Celloc | 0.4622  (0.002) | 0.4142  (0.001) | 0.3518  (0.003) | 0.2400  (0.002) | 0.1981  (0.002) | 0.1603  (0.002) | 0.1584  (0.002) | 0.1321  (0.001) | 0.0922  (0.005) |
|  | Cytospace | 0.5874  (0.007) | 0.5123  (0.006) | 0.3520  (0.006) | 0.5183  (0.007) | 0.4135  (0.006) | 0.3170  (0.005) | 0.3323  (0.006) | 0.3314  (0.007) | 0.1984  (0.006) |
|  | Tangram | 0.5585  (0.006) | 0.4650  (0.005) | 0.3401  (0.007) | 0.4872  (0.005) | 0.3999  (0.004) | 0.2964  (0.008) | 0.3491  (0.007) | 0.3083  (0.008) | 0.2000  (0.006) |
| Endothelial | Cell2Map | 0.9040  (0.009) | **0.9105**  (0.009) | 0.8769  (0.013) | 0.6506  (0.008) | 0.7070  (0.014) | 0.6255  (0.038) | 0.6148  (0.005) | 0.4658  (0.015) | 0.2766  (0.016) |
|  | Celloc | **0.9364**  (0.002) | 0.9020  (0.004) | **0.9320**  (0.007) | **0.8516**  (0.008) | **0.8555**  (0.008) | **0.7867**  (0.070) | **0.8176**  (0.005) | **0.7515**  (0.009) | **0.5929**  (0.051) |
|  | Cytospace | 0.6122  (0.005) | 0.5939  (0.006) | 0.4536  (0.007) | 0.5202  (0.006) | 0.6074  (0.006) | 0.2913  (0.031) | 0.4575  (0.007) | 0.3112  (0.008) | 0.2166  (0.009) |
|  | Tangram | 0.6180  (0.014) | 0.5704  (0.015) | 0.4396  (0.022) | 0.5124  (0.014) | 0.4231  (0.013) | 0.2884  (0.016) | 0.4281  (0.012) | 0.3068  (0.010) | 0.2049  (0.010) |
| Entorhinal | Cell2Map | **0.7855**  (0.020) | **0.6376**  (0.031) | **0.4509**  (0.005) | **0.7270**  (0.019) | **0.5589**  (0.019) | **0.4576**  (0.026) | **0.6916**  (0.007) | **0.4324**  (0.018) | **0.2909**  (0.032) |
|  | Celloc | 0.5398  (0.003) | 0.4837  (0.004) | 0.4388  (0.006) | 0.4415  (0.002) | 0.3833  (0.005) | 0.3621  (0.007) | 0.4186  (0.020) | 0.4118  (0.010) | 0.2804  (0.008) |
|  | Cytospace | 0.5332  (0.011) | 0.4873  (0.007) | 0.4239  (0.006) | 0.4533  (0.007) | 0.4363  (0.008) | 0.3108  (0.011) | 0.4247  (0.013) | 0.3352  (0.014) | 0.2733  (0.011) |
|  | Tangram | 0.5193  (0.012) | 0.4615  (0.013) | 0.3955  (0.018) | 0.4875  (0.011) | 0.4216  (0.013) | 0.3256  (0.013) | 0.4123  (0.014) | 0.3423  (0.011) | 0.2692  (0.012) |
| Ependyma | Cell2Map | **0.6037**  (0.013) | **0.5429**  (0.032) | **0.4929**  (0.006) | **0.5503**  (0.013) | **0.3791**  (0.008) | **0.3351**  (0.015) | **0.4412**  (0.003) | **0.3981**  (0.006) | **0.2836**  (0.008) |
|  | Celloc | 0.5709  (0.002) | 0.5210  (0.003) | 0.4870  (0.009) | 0.3520  (0.002) | 0.3064  (0.001) | 0.2212  (0.010) | 0.3027  (0.001) | 0.2334  (0.003) | 0.1362  (0.013) |
|  | Cytospace | 0.5710  (0.006) | 0.4872  (0.005) | 0.3934  (0.004) | 0.5424  (0.007) | 0.4311  (0.007) | 0.3287  (0.009) | 0.4332  (0.004) | 0.3475  (0.006) | 0.1924  (0.007) |
|  | Tangram | 0.5680  (0.013) | 0.4913  (0.012) | 0.3941  (0.013) | 0.5166  (0.008) | 0.4287  (0.007) | 0.3129  (0.009) | 0.4298  (0.006) | 0.3393  (0.007) | 0.2162  (0.008) |
| Fibroblast | Cell2Map | **0.8228**  (0.013) | **0.7535**  (0.013) | **0.6851**  (0.008) | 0.4478  (0.005) | **0.4889**  (0.017) | 0.4165  (0.020) | **0.5799**  (0.005) | 0.4213  (0.008) | **0.3697**  (0.021) |
|  | Celloc | 0.7593  (0.003) | 0.7001  (0.007) | 0.6429  (0.029) | **0.5886**  (0.006) | 0.4630  (0.026) | **0.4493**  (0.009) | 0.5575  (0.006) | **0.5000**  (0.009) | 0.3574  (0.023) |
|  | Cytospace | 0.5985  (0.014) | 0.5902  (0.011) | 0.3903  (0.008) | 0.5243  (0.013) | 0.4336  (0.009) | 0.3044  (0.013) | 0.4580  (0.012) | 0.3265  (0.009) | 0.2114  (0.019) |
|  | Tangram | 0.5867  (0.018) | 0.5239  (0.024) | 0.4282  (0.023) | 0.5213  (0.011) | 0.4205  (0.020) | 0.2950  (0.026) | 0.4298  (0.013) | 0.3336  (0.010) | 0.2058  (0.012) |
| Gad2 | Cell2Map | **0.6209**  (0.015) | 0.5240  (0.048) | 0.3882  (0.007) | **0.7316**  (0.021) | **0.5528**  (0.013) | **0.4257**  (0.010) | 0.4068  (0.008) | 0.2836  (0.025) | 0.2742  (0.029) |
|  | Celloc | 0.4385  (0.003) | 0.3976  (0.003) | 0.3527  (0.006) | 0.3100  (0.003) | 0.2762  (0.003) | 0.2498  (0.004) | 0.3332  (0.005) | 0.2958  (0.005) | 0.2181  (0.010) |
|  | Cytospace | 0.6122  (0.011) | **0.5981**  (0.008) | 0.4375  (0.006) | 0.5517  (0.010) | 0.5366  (0.011) | 0.4119  (0.012) | **0.5413**  (0.010) | **0.4915**  (0.011) | 0.3738  (0.014) |
|  | Tangram | 0.6190  (0.019) | 0.5688  (0.017) | **0.4759**  (0.022) | 0.5830  (0.014) | 0.5126  (0.013) | 0.4131  (0.023) | 0.5396  (0.014) | 0.4849  (0.016) | **0.3840**  (0.018) |
| Microglia | Cell2Map | **0.7886**  (0.011) | **0.7416**  (0.018) | **0.6626**  (0.008) | **0.4879**  (0.006) | **0.4304**  (0.011) | **0.2196**  (0.035) | **0.4484**  (0.004) | **0.2960**  (0.007) | **0.1496**  (0.005) |
|  | Celloc | 0.7655  (0.003) | 0.7329  (0.004) | 0.1741  (0.148) | 0.4767  (0.003) | 0.3671  (0.012) | 0.1802  (0.091) | 0.3416  (0.007) | 0.2329  (0.009) | 0.1336  (0.029) |
|  | Cytospace | 0.6008  (0.007) | 0.5144  (0.006) | 0.3124  (0.004) | 0.4575  (0.007) | 0.3244  (0.008) | 0.1688  (0.014) | 0.3245  (0.005) | 0.2353  (0.007) | 0.1037  (0.006) |
|  | Tangram | 0.5405  (0.009) | 0.4836  (0.009) | 0.3338  (0.005) | 0.4193  (0.010) | 0.3188  (0.010) | 0.1872  (0.009) | 0.3120  (0.005) | 0.2122  (0.006) | 0.1101  (0.008) |
| Mural | Cell2Map | **1.0000**  (0.000) | **1.0000**  (0.000) | 0.9835  (0.027) | **0.7103**  (0.013) | **0.6609**  (0.024) | 0.4246  (0.101) | **0.6128**  (0.009) | 0.4954  (0.022) | 0.1896  (0.028) |
|  | Celloc | **1.0000**  (0.000) | **1.0000**  (0.000) | **1.0000**  (0.000) | 0.3879  (0.054) | 0.6463  (0.305) | **0.9042**  (0.303) | 0.3084  (0.167) | **1.0000**  (0.000) | **0.9250**  (0.121) |
|  | Cytospace | 0.8127  (0.011) | 0.6738  (0.008) | 0.4516  (0.007) | 0.6574  (0.010) | 0.5375  (0.009) | 0.4114  (0.011) | 0.5915  (0.014) | 0.5150  (0.015) | 0.3178  (0.015) |
|  | Tangram | 0.7106  (0.040) | 0.6429  (0.059) | 0.4846  (0.054) | 0.6362  (0.032) | 0.5420  (0.027) | 0.3784  (0.041) | 0.5837  (0.034) | 0.4841  (0.047) | 0.3161  (0.044) |
| Neuro-  genesis | Cell2Map | **0.7331**  (0.018) | **0.6568**  (0.012) | **0.5308**  (0.003) | **0.5724**  (0.008) | **0.4554**  (0.020) | **0.2994**  (0.026) | **0.3216**  (0.004) | **0.2308**  (0.005) | **0.1312**  (0.012) |
|  | Celloc | 0.6396  (0.004) | 0.5922  (0.004) | 0.2371  (0.080) | 0.3362  (0.003) | 0.2680  (0.005) | 0.1347  (0.028) | 0.1876  (0.007) | 0.1508  (0.010) | 0.0821  (0.020) |
|  | Cytospace | 0.4934  (0.005) | 0.4105  (0.003) | 0.3533  (0.004) | 0.3478  (0.003) | 0.2717  (0.004) | 0.2185  (0.004) | 0.2267  (0.003) | 0.1376  (0.007) | 0.1154  (0.010) |
|  | Tangram | 0.4726  (0.010) | 0.3981  (0.008) | 0.2904  (0.009) | 0.3168  (0.005) | 0.2557  (0.007) | 0.1747  (0.004) | 0.2066  (0.004) | 0.1528  (0.006) | 0.0989  (0.004) |
| Oligo-  dendrocyte | Cell2Map | **1.0000**  (0.000) | **1.0000**  (0.000) | **1.0000**  (0.000) | 0.7147  (0.014) | 0.5585  (0.067) | 0.4471  (0.071) | 0.3865  (0.006) | 0.2253  (0.011) | 0.1122  (0.023) |
|  | Celloc | **1.0000**  (0.000) | **1.0000**  (0.000) | 0.9918  (0.017) | **0.8801**  (0.021) | **0.9023**  (0.024) | **0.9550**  (0.096) | **1.0000**  (0.000) | **1.0000**  (0.000) | **0.7060**  (0.112) |
|  | Cytospace | 0.5536  (0.012) | 0.4327  (0.010) | 0.3415  (0.012) | 0.3135  (0.011) | 0.2827  (0.010) | 0.1628  (0.011) | 0.1918  (0.010) | 0.1593  (0.013) | 0.0464  (0.006) |
|  | Tangram | 0.4918  (0.035) | 0.4658  (0.030) | 0.3067  (0.034) | 0.3234  (0.023) | 0.2361  (0.019) | 0.1513  (0.022) | 0.1826  (0.009) | 0.1582  (0.012) | 0.0628  (0.015) |
| Poly-  dendrocyte | Cell2Map | 0.4805  (0.023) | 0.3769  (0.014) | 0.3147  (0.003) | 0.3873  (0.011) | **0.4189**  (0.007) | 0.1491  (0.014) | 0.3108  (0.004) | **0.3433**  (0.008) | **0.2484**  (0.008) |
|  | Celloc | 0.3546  (0.003) | 0.3275  (0.001) | 0.2667  (0.004) | 0.1359  (0.001) | 0.1130  (0.001) | 0.1004  (0.002) | 0.0768  (0.001) | 0.0629  (0.001) | 0.0546  (0.001) |
|  | Cytospace | **0.6122**  (0.006) | **0.5288**  (0.005) | **0.4111**  (0.005) | **0.4723**  (0.004) | 0.4129  (0.006) | **0.2763**  (0.008) | **0.3612**  (0.006) | 0.3117  (0.007) | 0.1972  (0.008) |
|  | Tangram | 0.5703  (0.021) | 0.5040  (0.010) | 0.3499  (0.016) | 0.4657  (0.010) | 0.3796  (0.013) | 0.2558  (0.008) | 0.3594  (0.009) | 0.2885  (0.013) | 0.1830  (0.009) |
| Sic17ab | Cell2Map | **0.7141**  (0.024) | **0.5663**  (0.013) | **0.3992**  (0.005) | **0.6544**  (0.010) | **0.4851**  (0.011) | **0.2996**  (0.016) | **0.3893**  (0.005) | **0.3366**  (0.018) | **0.2402**  (0.010) |
|  | Celloc | 0.4782  (0.003) | 0.4216  (0.003) | 0.3669  (0.004) | 0.2761  (0.002) | 0.2405  (0.002) | 0.1899  (0.008) | 0.2047  (0.003) | 0.1613  (0.004) | 0.1150  (0.006) |
|  | Cytospace | 0.5325  (0.004) | 0.4637  (0.003) | 0.3256  (0.003) | 0.5067  (0.004) | 0.4032  (0.005) | 0.2901  (0.006) | 0.3717  (0.007) | 0.3208  (0.007) | 0.2248  (0.005) |
|  | Tangram | 0.5297  (0.011) | 0.4461  (0.013) | 0.3440  (0.016) | 0.4692  (0.012) | 0.3847  (0.008) | 0.2841  (0.005) | 0.3844  (0.011) | 0.3150  (0.008) | 0.2050  (0.011) |

**Table S13**. Runtime comparison (minutes) across methods and datasets.

| **Dataset** | **Cell2Map** | **Celloc** | **CytoSPACE** | **Tangram** |
| --- | --- | --- | --- | --- |
| Cerebellum (5 cells/spot) | ~ 4 min | ~5 min | ~8 min | ~3 min |
| Cerebellum (15 cells/spot) | ~ 4 min | ~6 min | ~10 min | ~4 min |
| Cerebellum (30 cells/spot) | ~ 4min | ~5 min | ~13 min | ~4 min |
| Hippocampus (5 cells/spot) | ~5 min | ~6 min | ~9 min | ~7 min |
| DCIS1 | ~2 min | ~4 min | ~12 min | ~5 min |
| DCIS2 | ~2 min | ~4 min | ~15 min | ~4 min |
| Breast cancer | ~2 min | ~4 min | ~20 min | ~3 min |

**Table S14**. Peak GPU memory usage (MiB) across methods and datasets.

| **Dataset** | **Cell2Map** | **Celloc** | **CytoSPACE** | **Tangram** |
| --- | --- | --- | --- | --- |
| Cerebellum (5cells/spot) | ~13,200 | ~12,400 | ~11,800 | ~8,100 |
| Hippocampus(5cells/spot) | ~12,800 | ~11,700 | ~10,000 | ~8,300 |
| DCIS1 | ~4,200 | ~5,100 | ~5,400 | ~5,200 |
| DCIS2 | ~5,000 | ~5,100 | ~5,200 | ~5,500 |
| Breast cancer | ~4,800 | ~4,300 | ~4,500 | ~4,000 |

**Table S15**. The results of ablation experiments on the simulated mouse hippocampus dataset with 5% noise and 5-cell-per-spot resolution with two different baselines.

| **Model** | | **PCA+ four-term objective** | **Autoencoder + four-term objective** | **Cell2Map** |
| --- | --- | --- | --- | --- |
| ACC | | 0.5656 | 0.5082 | **0.6848** |
| $Pr_{c2s}$ of 17 cell types | Astrocyte | 0.7763 | 0.7237 | **0.9309** |
|  | CA1 | 0.5000 | **0.7880** | 0.6285 |
|  | CA2CA3 | 0.6618 | 0.3041 | **0.7340** |
|  | Cajal Retzius | 0.5604 | 0.4815 | **0.6785** |
|  | Choroid | 0.6647 | **0.8392** | 0.5475 |
|  | Dentate | 0.5114 | 0.6045 | **0.6524** |
|  | Endothelial | 0.8179 | 0.5183 | **0.9040** |
|  | Entorhinal | 0.5219 | 0.6184 | **0.7855** |
|  | Ependyma | 0.5762 | 0.7585 | **0.6037** |
|  | Fibroblast | 0.7639 | 0.5517 | **0.8228** |
|  | Gad2 | 0.4775 | **0.7172** | 0.6209 |
|  | Microglia | 0.7801 | 0.2084 | **0.7886** |
|  | Mural | 1.0000 | 0.3224 | **1.0000** |
|  | Neurogenesis | 0.6022 | 0.4741 | **0.7331** |
|  | Oligodendrocyte | 0.8085 | 0.6331 | **1.0000** |
|  | Polydendrocyte | 0.5326 | **0.6549** | 0.4805 |
|  | Sic17ab | 0.5086 | 0.5714 | **0.7141** |

**Table S16**. the overlapping rate $R_{overlap}$ for tumor cells in three real datasets.

|  | Cell2Map | Celloc | CytoSPACE | Tangram |
| --- | --- | --- | --- | --- |
| DCIS2 for three tumor subclones | **0.7863** | 0.7481 | 0.2373 | 0.1967 |
| DCIS1 for tumor cells | **0.7656** | 0.5372 | 0.2767 | 0.0765 |
| Breast cancer for epithelial cells | **0.7592** | 0.7244 | 0.4209 | 0.2873 |

**Table S17.** Functional annotation of marker genes for tumor and epithelial cells obtained by using DCIS2 scRNA-seq reference data.

| **Cell Type** | **Gene** | **Function Summary** | **PMID** |
| --- | --- | --- | --- |
| Tumor | AGR3 | This gene encodes a member of the disulfide isomerase (PDI) family of endoplasmic reticulum (ER) proteins that catalyze protein folding and thiol-disulfide interchange reactions. The encoded protein has an N-terminal ER-signal sequence, a catalytically active thioredoxin domain, and a C-terminal ER-retention sequence. This gene is expressed in ciliated airway epithelial cells and, in mouse, plays a role in ciliary beat frequency in multiciliated cells. This gene is also over-expressed in breast, ovarian, and prostrate cancers. | 32724387, 31291711, 31611954, 35857928, 34462207 |
|  | FOXA1 | This gene encodes a member of the forkhead class of DNA-binding proteins. These hepatocyte nuclear factors are transcriptional activators for liver-specific transcripts such as albumin and transthyretin, and they also interact with chromatin. Similar family members in mice have roles in the regulation of metabolism and in the differentiation of the pancreas and liver. | 36245246, 29993139, 33417085, 27791031, 17671124, 17373880 |
|  | RHOC | This gene encodes a member of the Rho family of small GTPases, which cycle between inactive GDP-bound and active GTP-bound states and function as molecular switches in signal transduction cascades. Rho proteins promote reorganization of the actin cytoskeleton and regulate cell shape, attachment, and motility. The protein encoded by this gene is prenylated at its C-terminus, and localizes to the cytoplasm and plasma membrane. It is thought to be important in cell locomotion. Overexpression of this gene is associated with tumor cell proliferation and metastasis. Multiple alternatively spliced variants, encoding the same protein, have been identified. | 11839578, 15574779, 19421144, 16187229, 18478927, 19815594, 12939257, 16979889, 19269969, 17980029 |
|  | RAB25 | The protein encoded by this gene is a member of the RAS superfamily of small GTPases. The encoded protein is involved in membrane trafficking and cell survival. This gene has been found to be a tumor suppressor and an oncogene, depending on the context. Two variants, one protein-coding and the other not, have been found for this gene. | 19795443, 27259233, 26646320, 15502842, 32342915 |
|  | CD24 | This gene encodes a sialoglycoprotein that is expressed on mature granulocytes and B cells and modulates growth and differentiation signals to these cells. The precursor protein is cleaved to a short 32 amino acid mature peptide which is anchored via a glycosyl phosphatidylinositol (GPI) link to the cell surface. This gene was missing from previous genome assemblies, but is properly located on chromosome 6. Non-transcribed pseudogenes have been designated on chromosomes 1, 15, 20, and Y. Alternative splicing results in multiple transcript variants. | 26444008, 35048182, 34146197, 33427725, 30381446, 28418843, 27837613, 26617852 |
|  | FXYD3 | This gene belongs to a small family of FXYD-domain containing regulators of Na+/K+ ATPases which share a 35-amino acid signature sequence domain, beginning with the sequence PFXYD, and containing 7 invariant and 6 highly conserved amino acids. This gene encodes a cell membrane protein that may regulate the function of ion-pumps and ion-channels. This gene may also play a role in tumor progression. Alternative splicing results in multiple transcript variants encoding distinct isoforms. | 14745797, 19571376, 7970700, 37966117, 26740212, 30206184, |
|  | AGR2 | This gene encodes a member of the disulfide isomerase (PDI) family of endoplasmic reticulum (ER) proteins that catalyze protein folding and thiol-disulfide interchange reactions. The encoded protein has an N-terminal ER-signal sequence, a catalytically active thioredoxin domain, and a C-terminal ER-retention sequence. This protein plays a role in cell migration, cellular transformation and metastasis and is as a p53 inhibitor. As an ER-localized molecular chaperone, it plays a role in the folding, trafficking, and assembly of cysteine-rich transmembrane receptors and the cysteine-rich intestinal gylcoprotein mucin. This gene has been implicated in inflammatory bowel disease and cancer progression. | 35857928, 25666661, 37197416, 16551856, 9790916 |
|  | CLDN4 | The protein encoded by this intronless gene belongs to the claudin family. Claudins are integral membrane proteins that are components of the epithelial cell tight junctions, which regulate movement of solutes and ions through the paracellular space. This protein is a high-affinity receptor for Clostridium perfringens enterotoxin (CPE) and may play a role in internal organ development and function during pre- and postnatal life. This gene is deleted in Williams-Beuren syndrome, a neurodevelopmental disorder affecting multiple systems. | 33147053, 32086991, 30808546, 30025999, 29509190, 29482498, 28559303, 26566278 |
| Epithelial | N4BP2L1 | N4BP2L1 (NEDD4 Binding Protein 2 Like 1) is a Protein Coding gene. Gene Ontology (GO) annotations related to this gene include kinase activity. An important paralog of this gene is N4BP2. | 38859961, 41542042 |
|  | SON | This gene encodes a protein that contains multiple simple repeats. The encoded protein binds RNA and promotes pre-mRNA splicing, particularly of transcripts with poor splice sites. The protein also recognizes a specific DNA sequence found in the human hepatitis B virus (HBV) and represses HBV core promoter activity. There is a pseudogene for this gene on chromosome 1. Alternative splicing results in multiple transcript variants. | 39313574, 33095160 |
|  | ANKRD30A | This gene encodes a DNA-binding transcription factor that is uniquely expressed in mammary epithelium and the testis. Altered expression levels have been associated with breast cancer progression. | 29116378, 27863482, 26415367, 33005030, |
|  | CCNL2 | The protein belongs to the cyclin family. Through its interaction with several proteins, such as RNA polymerase II, splicing factors, and cyclin-dependent kinases, this protein functions as a regulator of the pre-mRNA splicing process, as well as in inducing apoptosis by modulating the expression of apoptotic and antiapoptotic proteins. Alternatively spliced transcript variants encoding different isoforms have been described for this gene. | 36238849, 33896262, 35784572 |
|  | CDK5RAP3 | This gene encodes a protein that has been reported to function in signaling pathways governing transcriptional regulation and cell cycle progression. It may play a role in tumorigenesis and metastasis. A pseudogene of this gene is located on the long arm of chromosome 20. Alternative splicing results in multiple transcript variants that encode different isoforms. | 35053516 |
|  | DDX17 | DEAD box proteins, characterized by the conserved motif Asp-Glu-Ala-Asp (DEAD), are putative RNA helicases. They are implicated in a number of cellular processes involving alteration of RNA secondary structure. This gene encodes a DEAD box protein, which is an ATPase activated by a variety of RNA species, but not by dsDNA. This protein, and that encoded by DDX5 gene, are more closely related to each other than to any other member of the DEAD box family. This gene can encode multiple isoforms due to both alternative splicing and the use of alternative translation initiation codons, including a non-AUG (CUG) start codon. | 19718048, 26569340 |
|  | RSRP1 | Involved in spliceosomal complex assembly. Located in nucleus. | 26209438 |
|  | PNISR | Enables RNA binding activity. Located in cytosol; nuclear speck; and plasma membrane. | 14578391, 23299472 |

**Table S18.** Functional annotation of marker genes for tumor and natural killer T cells (NK/T) obtained by using DCIS1 scRNA-seq reference data.

| **Cell Type** | **Gene** | **Functional Description** | **PMID** |
| --- | --- | --- | --- |
| Tumor | ERBB2 | This gene encodes a member of the epidermal growth factor (EGF) receptor family of receptor tyrosine kinases. This protein has no ligand binding domain of its own and therefore cannot bind growth factors. However, it does bind tightly to other ligand-bound EGF receptor family members to form a heterodimer, stabilizing ligand binding and enhancing kinase-mediated activation of downstream signalling pathways, such as those involving mitogen-activated protein kinase and phosphatidylinositol-3 kinase. Allelic variations at amino acid positions 654 and 655 of isoform a (positions 624 and 625 of isoform b) have been reported, with the most common allele, Ile654/Ile655, shown here. Amplification and/or overexpression of this gene has been reported in numerous cancers, including breast and ovarian tumors. Alternative splicing results in several additional transcript variants, some encoding different isoforms and others that have not been fully characterized. | 9797688, 20371687, 11106082, 11304787, 9499914, 30621632, 28178722, 27576528, 27284958, 20961995, 20371687, 16883574, 16963728 |
|  | PSMD3 | The 26S proteasome is a multicatalytic proteinase complex with a highly ordered structure composed of 2 complexes, a 20S core and a 19S regulator. Proteasomes are distributed throughout eukaryotic cells at a high concentration and cleave peptides in an ATP/ubiquitin-dependent process in a non-lysosomal pathway. This gene encodes a member of the proteasome subunit S3 family. | 33742100, 31013812 |
|  | MIEN1 | Involved in negative regulation of apoptotic process; positive regulation of cell migration; and positive regulation of filopodium assembly. Located in cytoplasmic side of plasma membrane and cytosol. | 28198505, 21068479, 30286132 |
|  | TOB1 | This gene encodes a member of the transducer of erbB-2 /B-cell translocation gene protein family. Members of this family are anti-proliferative factors that have the potential to regulate cell growth. The encoded protein may function as a tumor suppressor. Alternate splicing results in multiple transcript variants. | 33786619, 32307083, 26165839, 23589165, 26377249, |
|  | PHB1 | This gene is evolutionarily conserved, and its product is proposed to play a role in human cellular senescence and tumor suppression. Antiproliferative activity is reported to be localized to the 3' UTR, which is proposed to function as a trans-acting regulatory RNA. Several pseudogenes of this gene have been identified. Alternative splicing results in multiple transcript variants. | 11377649, 37957856, 40816542, 31614178 |
|  | MRPL27 | Mammalian mitochondrial ribosomal proteins are encoded by nuclear genes and help in protein synthesis within the mitochondrion. Mitochondrial ribosomes (mitoribosomes) consist of a small 28S subunit and a large 39S subunit. They have an estimated 75% protein to rRNA composition compared to prokaryotic ribosomes, where this ratio is reversed. | 35739158, 39859078, 38617541 |
|  | HOXB2 | This gene is a member of the Antp homeobox family and encodes a nuclear protein with a homeobox DNA-binding domain. It is included in a cluster of homeobox B genes located on chromosome 17. The encoded protein functions as a sequence-specific transcription factor that is involved in development. Increased expression of this gene is associated with pancreatic cancer. | 38322121, 34062258, 21672623 |
|  | COPRS | Enables histone binding activity. Involved in chromatin remodeling. Located in cytosol; nucleoplasm; and plasma membrane. | 37314216, 28977470 |
| NK/T | PTPRC | The protein encoded by this gene is a member of the protein tyrosine phosphatase (PTP) family. PTPs are known to be signaling molecules that regulate a variety of cellular processes including cell growth, differentiation, mitosis, and oncogenic transformation. This PTP contains an extracellular domain, a single transmembrane segment and two tandem intracytoplasmic catalytic domains, and thus is classified as a receptor type PTP. This PTP has been shown to be an essential regulator of T- and B-cell antigen receptor signaling. It functions through either direct interaction with components of the antigen receptor complexes, or by activating various Src family kinases required for the antigen receptor signaling. This PTP also suppresses JAK kinases, and thus functions as a regulator of cytokine receptor signaling. Alternatively spliced transcripts variants of this gene, which encode distinct isoforms, have been reported. | 37388747, 30825183, 25267515, 22626783 |
|  | CD3D | The protein encoded by this gene is part of the T-cell receptor/CD3 complex (TCR/CD3 complex) and is involved in T-cell development and signal transduction. The encoded membrane protein represents the delta subunit of the CD3 complex, and along with four other CD3 subunits, binds either TCR alpha/beta or TCR gamma/delta to form the TCR/CD3 complex on the surface of T-cells. Defects in this gene are a cause of severe combined immunodeficiency autosomal recessive T-cell-negative/B-cell-positive/NK-cell-positive (SCIDBNK). Two transcript variants encoding different isoforms have been found for this gene. Other variants may also exist, but the full-length natures of their transcripts has yet to be defined. | 33350431, 21670311 |
|  | CD3E | The protein encoded by this gene is the CD3-epsilon polypeptide, which together with CD3-gamma, -delta and -zeta, and the T-cell receptor alpha/beta and gamma/delta heterodimers, forms the T-cell receptor-CD3 complex. This complex plays an important role in coupling antigen recognition to several intracellular signal-transduction pathways. The genes encoding the epsilon, gamma and delta polypeptides are located in the same cluster on chromosome 11. The epsilon polypeptide plays an essential role in T-cell development. Defects in this gene cause immunodeficiency. This gene has also been linked to a susceptibility to type I diabetes in women. | 39791074, 28537889, 33747959, 30340146 |
|  | CD3G | The protein encoded by this gene is the CD3-gamma polypeptide, which together with CD3-epsilon, -delta and -zeta, and the T-cell receptor alpha/beta and gamma/delta heterodimers, forms the T-cell receptor-CD3 complex. This complex plays an important role in coupling antigen recognition to several intracellular signal-transduction pathways. The genes encoding the epsilon, gamma and delta polypeptides are located in the same cluster on chromosome 11. Defects in this gene are associated with T cell immunodeficiency. | 25760691, 21670311 |
|  | CD2 | The protein encoded by this gene is a surface antigen found on all peripheral blood T-cells. The encoded protein interacts with LFA3 (CD58) on antigen presenting cells to optimize immune recognition. A locus control region (LCR) has been found in the 3' flanking sequence of this gene. | 28409241, 33968066, 40859981, 37666207 |
|  | TRAC | T cell receptors recognize foreign antigens which have been processed as small peptides and bound to major histocompatibility complex (MHC) molecules at the surface of antigen presenting cells (APC). Each T cell receptor is a dimer consisting of one alpha and one beta chain or one delta and one gamma chain. In a single cell, the T cell receptor loci are rearranged and expressed in the order delta, gamma, beta, and alpha. This region represents the germline organization of the T cell receptor alpha and delta loci. Both the alpha and delta loci include V (variable), J (joining), and C (constant) segments and the delta locus also includes diversity (D) segments. The delta locus is situated within the alpha locus, between the alpha V and J segments. During T cell development, the delta chain is synthesized by a recombination event at the DNA level joining a D segment with a J segment; a V segment is then joined to the D-J gene. The alpha chain is synthesized by recombination joining a single V segment with a J segment. For both chains, the C segment is later joined by splicing at the RNA level. Recombination of many different V segments with several J segments provides a wide range of antigen recognition. Additional diversity is attained by junctional diversity, resulting from the random additional of nucleotides by terminal deoxynucleotidyltransferase. Five variable segments can be used in either alpha or delta chains and are described by TRAV/DV symbols. Several V and J segments of the alpha locus are known to be incapable of encoding a protein and are considered pseudogenes. | 39271844, 35941372 |
|  | TRBC1 | Predicted to be involved in T cell receptor signaling pathway; adaptive immune response; and alpha-beta T cell activation. Predicted to be located in plasma membrane. Predicted to be part of alpha-beta T cell receptor complex. | 39528665, 38538786 |
|  | TRBC2 | Predicted to be involved in T cell receptor signaling pathway; adaptive immune response; and alpha-beta T cell activation. Located in plasma membrane. Part of alpha-beta T cell receptor complex. | 41429932, 40691137 |

**Table S19.** Functional annotation of marker genes for epithelial cells obtained by using scRNA-seq reference data of HER2+ FFPE breast cancer.

| **Cell Type** | **Gene** | **Functional Description** | **PMID** |
| --- | --- | --- | --- |
| Epithelial | AZGP1 | Predicted to enable RNA nuclease activity and protein transmembrane transporter activity. Involved in cell adhesion and detection of chemical stimulus involved in sensory perception of bitter taste. Located in extracellular space. Implicated in prostate carcinoma. Biomarker of several diseases, including gastrointestinal system cancer (multiple); kidney failure (multiple); liver cirrhosis; lung adenocarcinoma; and obesity. | 37927217, 38349455, 24114735, 20683033, 38888911, 23242172 |
|  | WFDC2 | This gene encodes a protein that is a member of the WFDC domain family. The WFDC domain, or WAP Signature motif, contains eight cysteines forming four disulfide bonds at the core of the protein, and functions as a protease inhibitor in many family members. This gene is expressed in pulmonary epithelial cells, and was also found to be expressed in some ovarian cancers. The encoded protein is a small secretory protein, which may be involved in sperm maturation. | 34422565, 30004048, 27192502, 40190530, 34994942, 27983524, 21115941 |
|  | TACSTD2 | This intronless gene encodes a carcinoma-associated antigen. This antigen is a cell surface receptor that transduces calcium signals. Mutations of this gene have been associated with gelatinous drop-like corneal dystrophy. | 37675806, 35882754, 36153494, 40516178, 40624355, 40806559 |
|  | TM4SF1 | The protein encoded by this gene is a member of the transmembrane 4 superfamily, also known as the tetraspanin family. Most of these members are cell-surface proteins that are characterized by the presence of four hydrophobic domains. The proteins mediate signal transduction events that play a role in the regulation of cell development, activation, growth and motility. This encoded protein is a cell surface antigen and is highly expressed in different carcinomas. | 26464650, 29987050, 29384939, 35153775, 33016133, 38251858 |
|  | EMP1 | Involved in apoptotic process and bleb assembly. Located in plasma membrane. | 41458590, 40016223, 32908789, 29867202 |
|  | AK5 | This gene encodes a member of the adenylate kinase family, which is involved in regulating the adenine nucleotide composition within a cell by catalyzing the reversible transfer of phosphate groups among adenine nucleotides. This member is related to the UMP/CMP kinase of several species. It is located in the cytosol and expressed exclusively in brain. | 39655021, 29928444 |
|  | FXYD3 | This gene belongs to a small family of FXYD-domain containing regulators of Na+/K+ ATPases which share a 35-amino acid signature sequence domain, beginning with the sequence PFXYD, and containing 7 invariant and 6 highly conserved amino acids. This gene encodes a cell membrane protein that may regulate the function of ion-pumps and ion-channels. This gene may also play a role in tumor progression. | 7836447, 19571376, 37966117, 26740212, 30206184 |
| Epithelial | TPM1 | This gene is a member of the tropomyosin family of highly conserved, widely distributed actin-binding proteins involved in the contractile system of striated and smooth muscles and the cytoskeleton of non-muscle cells. Tropomyosin is composed of two alpha-helical chains arranged as a coiled-coil. It is polymerized end to end along the two grooves of actin filaments and provides stability to the filaments. Mutations in this gene are associated with type 3 familial hypertrophic cardiomyopathy and dilated cardiomyopathy 1Y. | 26171250, 29221121, 12213212, 27130446, 35600659 |

**Table S20.** Functional annotation of marker genes for tumor and NK/T cells obtained by using DCIS1 scRNA-seq reference data.

| **Cell Type** | **Gene** | **Functional Description** | **PMID** |
| --- | --- | --- | --- |
| Tumor | HOXB3 | This gene is a member of the Antp homeobox family and encodes a nuclear protein with a homeobox DNA-binding domain. It is included in a cluster of homeobox B genes located on chromosome 17. The encoded protein functions as a sequence-specific transcription factor that is involved in development. Increased expression of this gene is associated with a distinct biologic subset of acute myeloid leukemia (AML). | 28075453, 22705304, 33240685, 32147544, 33138748 |
|  | SIX1 | The protein encoded by this gene is a homeobox protein that is similar to the Drosophila 'sine oculis' gene product. This gene is found in a cluster of related genes on chromosome 14 and is thought to be involved in limb development. Defects in this gene are a cause of autosomal dominant deafness type 23 (DFNA23) and branchiootic syndrome type 3 (BOS3). | 38031089, 34711117, 33293473, 26408179, 24438019, 24184484, 22466647, 40715465, |
|  | SOX11 | This intronless gene encodes a member of the SOX (SRY-related HMG-box) family of transcription factors involved in the regulation of embryonic development and in the determination of the cell fate. The encoded protein may act as a transcriptional regulator after forming a protein complex with other proteins. The protein may function in the developing nervous system and play a role in tumorigenesis. | 38760444, 33536579, 32043610, 26894864, 25041022, 32909943 |
|  | ONECUT2 | This gene encodes a member of the onecut family of transcription factors, which are characterized by a cut domain and an atypical homeodomain. The protein binds to specific DNA sequences and stimulates expression of target genes, including genes involved in melanocyte and hepatocyte differentiation. | 31118200, 38819630, 41222115 |
|  | EPN3 | Predicted to enable clathrin binding activity and phospholipid binding activity. Predicted to be involved in endocytosis. Located in several cellular components, including clathrin-coated vesicle; cytoplasmic side of plasma membrane; and perinuclear region of cytoplasm. | 27926932, 34779498, 32541686 |
|  | PRR15L | PRR15L (Proline Rich 15 Like) is a Protein Coding gene. Diseases associated with PRR15L include Penis Squamous Cell Carcinoma and Entropion. Among its related pathways is Pancreatic cancer subtypes. | 37072408, 36157240, 28380025, 34395436 |
|  | QPRT | This gene encodes a key enzyme in catabolism of quinolinate, an intermediate in the tryptophan-nicotinamide adenine dinucleotide pathway. Quinolinate acts as a most potent endogenous exitotoxin to neurons. Elevation of quinolinate levels in the brain has been linked to the pathogenesis of neurodegenerative disorders such as epilepsy, Alzheimer's disease, and Huntington's disease. Alternative splicing results in multiple transcript variants. | 37723185, 32716908, 35251175, 35345651, 40888212 |
|  | ST8SIA6-AS1 | ST8SIA6-AS1 (ST8SIA6 Antisense RNA 1) is an RNA Gene, and is affiliated with the lncRNA class. Diseases associated with ST8SIA6-AS1 include Cholangiocarcinoma and Lung Cancer Susceptibility 3. | 31784750, 33037712, 35730485, 31286138 |
| NK/T | CD3D | The protein encoded by this gene is part of the T-cell receptor/CD3 complex (TCR/CD3 complex) and is involved in T-cell development and signal transduction. The encoded membrane protein represents the delta subunit of the CD3 complex, and along with four other CD3 subunits, binds either TCR alpha/beta or TCR gamma/delta to form the TCR/CD3 complex on the surface of T-cells. Defects in this gene are a cause of severe combined immunodeficiency autosomal recessive T-cell-negative/B-cell-positive/NK-cell-positive (SCIDBNK). | 33350431, 21670311 |
|  | CD3E | The protein encoded by this gene is the CD3-epsilon polypeptide, which together with CD3-gamma, -delta and -zeta, and the T-cell receptor alpha/beta and gamma/delta heterodimers, forms the T-cell receptor-CD3 complex. This complex plays an important role in coupling antigen recognition to several intracellular signal-transduction pathways. The genes encoding the epsilon, gamma and delta polypeptides are located in the same cluster on chromosome 11. The epsilon polypeptide plays an essential role in T-cell development. Defects in this gene cause immunodeficiency. This gene has also been linked to a susceptibility to type I diabetes in women. | 39791074, 28537889, 33747959, 30340146 |
|  | PTPRC | The protein encoded by this gene is a member of the protein tyrosine phosphatase (PTP) family. PTPs are known to be signaling molecules that regulate a variety of cellular processes including cell growth, differentiation, mitosis, and oncogenic transformation. This PTP contains an extracellular domain, a single transmembrane segment and two tandem intracytoplasmic catalytic domains, and thus is classified as a receptor type PTP. This PTP has been shown to be an essential regulator of T- and B-cell antigen receptor signaling. It functions through either direct interaction with components of the antigen receptor complexes, or by activating various Src family kinases required for the antigen receptor signaling. This PTP also suppresses JAK kinases, and thus functions as a regulator of cytokine receptor signaling. Alternatively spliced transcripts variants of this gene, which encode distinct isoforms, have been reported. | 37388747, 30825183, 25267515, 22626783 |
|  | IL32 | This gene encodes a member of the cytokine family. The protein contains a tyrosine sulfation site, 3 potential N-myristoylation sites, multiple putative phosphorylation sites, and an RGD cell-attachment sequence. Expression of this protein is increased after the activation of T-cells by mitogens or the activation of NK cells by IL-2. This protein induces the production of TNFalpha from macrophage cells. Alternate transcriptional splice variants, encoding different isoforms, have been characterized. | 30391782, 28470472, 25100201, 30615365, 25449783, 18501120, 25435980 |
|  | CD2 | The protein encoded by this gene is a surface antigen found on all peripheral blood T-cells. The encoded protein interacts with LFA3 (CD58) on antigen presenting cells to optimize immune recognition. A locus control region (LCR) has been found in the 3' flanking sequence of this gene. | 28409241, 33968066, 40859981, 37666207 |
|  | CD3G | The protein encoded by this gene is the CD3-gamma polypeptide, which together with CD3-epsilon, -delta and -zeta, and the T-cell receptor alpha/beta and gamma/delta heterodimers, forms the T-cell receptor-CD3 complex. This complex plays an important role in coupling antigen recognition to several intracellular signal-transduction pathways. The genes encoding the epsilon, gamma and delta polypeptides are located in the same cluster on chromosome 11. Defects in this gene are associated with T cell immunodeficiency. | 25760691, 21670311 |
|  | FYB1 | The protein encoded by this gene is an adapter for the FYN protein and LCP2 signaling cascades in T-cells. The encoded protein is involved in platelet activation and controls the expression of interleukin-2. Three transcript variants encoding different isoforms have been found for this gene. | 38334954, 37767202 |
|  | IL7R | The protein encoded by this gene is a receptor for interleukin 7 (IL7). The function of this receptor requires the interleukin 2 receptor, gamma chain (IL2RG), which is a common gamma chain shared by the receptors of various cytokines, including interleukins 2, 4, 7, 9, and 15. This protein has been shown to play a critical role in V(D)J recombination during lymphocyte development. Defects in this gene may be associated with severe combined immunodeficiency (SCID). Alternatively spliced transcript variants have been found. | 38104284, 31998790, 28964592, 38638044, 32659700, 17546596, 38104284 |

**Figure S1.** Additional marker genes of tumor and normal epithelial cells in ductal carcinoma DCIS2 sample. **a.** Volcano plot showing differential gene expression between tumor cells and all other cell populations. **b.** Volcano plot comparing normal epithelial cells with all other cell populations. **c.** Spatial expression of four additional tumor markers (CLDN4, CD24, FXYD3, AGR2) and four normal epithelial markers (DDX17, ANKRD30A, CCNL2, PNISR). Color intensity represents normalized gene expression levels.

**Figure S2.** Additional marker genes of tumor and NK/T cells in ductal carcinoma DCIS1 sample. **a.** Volcano plot showing differential gene expression between tumor cells and all other cell populations. **b.** Volcano plot comparing NK/T cells with all other cell populations. **c.** Spatial expression of four additional tumor markers (PHB1, MRPL27, COPRS, HOXB2) and four NK/T cell markers (CD2, TRAC, TRBC1, TRBC2). Color intensity represents normalized gene expression levels.

**Figure S3.** Cell-to-spot mapping of a MI SRT and scRNA-seq datasets. **a.** H&E-stained image of a 10X Visium MI SRT tissue section, with histologically annotated fibrotic regions outlined in red lines. **b.** UMAP plot of the scRNA-seq dataset annotated with major cell types. c. Spatial mapping results of all mapped cell types (top), MP (median) and FB (bottom) cells. CM: Cardiomyocyte, ENDO: Endothelial cell, FB: Fibroblast, MP: Macrophage, SMC: smooth muscle cell.

**Figure S4.** Marker genes of epithelial cells in breast cancer. **a.** Volcano plot showing differential gene expression between epithelial cells and all other cell populations by using the HER2+ FFPE scRNA-seq data. **b.** Spatial expression of eight markers of epithelial cells. Color intensity represents normalized gene expression levels.

**Figure S5.** Marker genes of NK/T cells in breast cancer. **a.** Volcano plot showing differential gene expression between NK/T cells and all other cell populations using the DCIS1 scRNA-seq data. **b.** Spatial expression of eight markers of NK/T cells. Color intensity represents normalized gene expression levels.

**Figure S6.** Marker genes of tumor cells in breast cancer. **a.** Volcano plot showing differential gene expression between tumor cells and all other cell populations using the DCIS1 scRNA-seq data. **b.** Spatial expression of eight markers of tumor cells. Color intensity represents normalized gene expression levels.
